# Small, open-source text-embedding models as substitutes to OpenAI models for gene analysis

**DOI:** 10.1016/j.csbj.2025.07.053

**Published:** 2025-08-06

**Authors:** Dailin Gan, Jun Li

**Affiliations:** Department of Applied and Computational Mathematics and Statistics, University of Notre Dame, Notre Dame, IN, USA

## Abstract

While foundation transformer-based models developed for gene expression data analysis can be costly to train and operate, a recent approach known as GenePT offers a low-cost and highly efficient alternative. GenePT utilizes OpenAI's text-embedding function to encode background information, which is in textual form, about genes. However, the closed-source, online nature of OpenAI's text-embedding service raises concerns regarding data privacy, among other issues. In this paper, we explore the possibility of replacing OpenAI's models with open-source transformer-based text-embedding models. We identified ten models from Hugging Face that are small in size, easy to install, and light in computation. Across all four gene classification tasks we considered, some of these models have outperformed OpenAI's, demonstrating their potential as viable, or even superior, alternatives. Additionally, we find that fine-tuning these models often does not lead to significant improvements in performance.

## Graphical abstract

**Figure:**
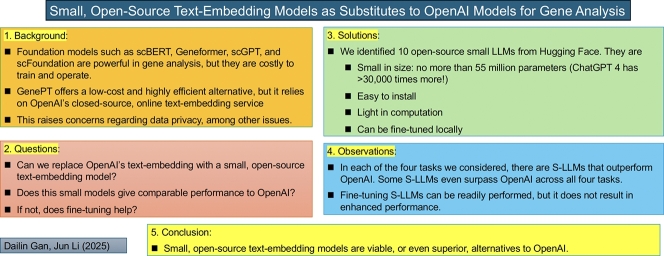

## 1 Introduction

The integration of large language models (LLMs), particularly transformer models [1], into gene-expression data analysis has generated substantial interest due to their improved predictive capabilities across various applications (see, e.g., [2], [3], [4], [5], [6], [7], [8] for a review). Foundation models like scBERT [9], Geneformer [10], scGPT [11], and scFoundation [12] are trained on extensive collections of single-cell RNA-seq data that include thousands of datasets and millions of cells. This training process is designed to capture intricate information about genes and their interactions. Subsequently, these models are fine-tuned on much smaller, user-specific datasets tailored to specific applications. However, the development of foundation models from scratch necessitates significant resources. The extensive data collection and computational demands often make this process prohibitively expensive for most research groups. Additionally, deploying and fine-tuning these models for personalized applications presents challenges related to hardware requirements, software compatibility, and programming expertise [13].

The recently developed GenePT [14] introduces an innovative approach that eliminates the necessity of training foundation models from scratch. Utilizing OpenAI's text-embedding function, this method transforms standard NCBI gene descriptions [15] into embeddings—dense numeric vectors that effectively encapsulate the textual content [16]. These embeddings comprehensively capture gene functions and their interactions as detailed in NCBI descriptions, offering a novel way to summarize and utilize existing knowledge about genes. These embeddings are directly employed as inputs for various machine learning methods, such as logistic regression and random forests, across diverse applications, such as classifying genes into drug-sensitive or drug-insensitive categories. The GenePT approach has been shown to achieve accuracy comparable to, or even surpassing, that of gene-expression foundation models in various gene-classification tasks [14].

The advantages of the GenePT approach are significant. It leverages OpenAI's publicly accessible text-embedding function, eliminating the need for resource-intensive training of gene-expression foundation models. Additionally, this method is highly generalizable and can be extended to numerous other applications; OpenAI's tool is capable of embedding descriptions of various biological entities, not limited to genes. Consequently, this approach is poised for broad adoption in the near future. Indeed, text-embedding plays a crucial role in natural language processing, where it is widely used in key applications such as text retrieval and translation, among others (see, e.g., [17] for a review).

However, relying on OpenAI's text-embedding can also impose limitations on GenePT's applicability. OpenAI's embedding is an online service that requires users to send text to the OpenAI server and retrieve the embeddings from it. This reliance not only raises concerns about internet connectivity and security but also, and more critically, about the privacy of the uploaded text. While privacy might not be a significant concern for NCBI's descriptions of genes, it becomes crucial if researchers wish to include their own insights, research results, or descriptions of other entities that are intended to remain confidential. The privacy issues associated with online LLMs have gained significant attention (see, e.g., [18], [19]); a recent study [20] reports that “all the methods leak query data,” and concludes that “to achieve truly privacy-preserving LLM adaptations that yield high performance and more privacy at lower costs, taking into account current methods and models, one should use open LLMs.” Additionally, using an online service raises other potential concerns, such as the reproducibility of results [21]. Furthermore, OpenAI charges a per-token fee for text embedding.

In this paper, we aim to address these limitations by replacing OpenAI's service with open-source, transformer-based text-embedding models that can be installed and run locally. To ensure manageability for regular users with limited computational resources, we limit the size of these models to less than 100 million parameters. We refer to these as “small LLMs,” “small language models,” or SLMs for short. It is important to note that SLMs are general-purpose text-embedding models designed to convert natural-language text into embeddings, and they are not the gene-expression foundation models discussed earlier. We will test these SLMs across the four gene-property prediction tasks discussed in the GenePT paper to determine if they perform comparably to GenePT's approach, which relies on OpenAI's embedding model. Specifically, we will employ logistic regression or random forest on the embeddings generated by SLMs or OpenAI's model, and compare the accuracy of the predictions.

In the broader context of natural language processing, the performance of various embedding tools has been systematically assessed through the Massive Text Embedding Benchmark (MTEB) [22], which includes 58 datasets across 112 languages from eight embedding tasks, such as document classification, clustering, and retrieval. On average across these datasets, OpenAI's embedding tool outperforms most open-source alternatives. However, some open-source models can perform just as well, or even better than, OpenAI's tools in specific tasks. Therefore, it will be interesting to see whether the SLMs we have selected can match the performance of OpenAI's embeddings in gene analysis.

If the SLMs cannot compete, there is a potential remedy: fine-tuning the SLMs. Specifically, instead of using the static text embeddings provided by the SLM as inputs for a separate classifier such as logistic regression or random forest, we could integrate an additional layer into the SLM, transforming it into a combined embedding and classification system. This integrated system would then be fine-tuned as a whole, allowing the embeddings to evolve during training. It is important to note that this fine-tuning process involves only minor adjustments to the weights in the pre-trained transformer model, resulting in a computational burden significantly lower than that of training a SLM from scratch. It is also worth noting that the capability to fine-tune is not available with OpenAI's embedding function, which is closed-source online service.

## 2 Results

### 2.1 Settings of the comparisons

Our comparisons focus on the four gene-property prediction tasks considered in the GenePT paper [23]. For the remainder of this paper, we will refer to these tasks as Task 1 through Task 4, respectively. Each of these tasks is a two-class classification problem, classifying genes into categories such as dosage-sensitive or dosage-insensitive transcription factors. Table 1 details the two classes and the sample size for each class. These datasets were curated, pre-processed, and made available by [10].

**Table 1:** Datasets and the number of samples in each class.

	Class 1	**Table:** Sample Size in Class 1	Class 2	**Table:** Sample Size in Class 2
**Task 1**	**Table:** Long-range transcription factors	46	**Table:** Short-range transcription factors	130
**Task 2**	**Table:** Dosage-sensitive transcription factors	122	**Table:** Dosage-insensitive transcription factors	368
**Task 3**	Bivalent genes	107	**Table:** Lys4-only-methylated genes	80
**Task 4**	Bivalent genes	107	**Table:** Non-methylated genes	42

For the gene descriptions, we utilized all human genes from the genome assembly version GRCh38.111, obtained from the Ensembl database [24]. Following the procedures outlined in the GenePT paper [23], we extracted descriptions from the summary section of the NCBI Gene database, after removing hyperlinks and date information. These cleaned descriptions were then used as input to generate text embeddings.

Hugging Face [25] offers a wide range of publicly available transformer-based text embedding models. These models vary greatly in size, measured by the number of parameters, and are categorized into five groups: fewer than 100 million, 100 million to 250 million, 250 million to 500 million, 500 million to 1 billion, and over 1 billion. Models on Hugging Face are systematically evaluated using the MTEB.

We selected ten models with fewer than 100 million parameters that ranked highest on the Hugging Face Leaderboard (https://huggingface.co/spaces/mteb/leaderboard_legacy, as of December 9th, 2024) for the “classification” embedding task in English. The models chosen include GIST-small-Embedding-v0 [26], NoInstruct-small-Embedding-v0 [27], stella-base-en-v2 [28], bge-small-en-v1.5 [29], e5-small-v2 [30], GIST-all-MiniLM-L6-v2 [26], gte-small [31], MedEmbed-small-v0.1 [32], e5-small [30], and gte-tiny [33]. A summary of these ten models is presented in Table 2, which includes the number of parameters, memory usage, average performance on MTEB classification datasets, and the embedding dimension each model produces. Notably, all of these SLMs contain no more than 55 million parameters. For context, OpenAI's GPT-3 model contains 175 billion parameters—over a thousand times larger than our SLMs—and GPT-4 has 1.76 trillion parameters. These SLMs require a minimal amount of memory and can be easily installed and operated on a regular laptop computer. Except for stella-base-en-v2, which produces 768-dimensional embeddings, all other SLMs generate 384-dimensional embeddings.

**Table 2:** Information about the ten SLMs we use; more detailed information for each model can be found on the MTEB Hugging Face Leaderboard (https://huggingface.co/spaces/mteb/leaderboard_legacy).

Models	Model parameters (million)	Memory Usage (GB)	MTEB Average Performance	Embedding dimensions
GIST-small-Embedding-v0	33	0.12	76.1	384
NoInstruct-small-Embedding-v0	33	0.12	76.0	384
stella-base-en-v2	55	0.20	75.3	768
e5-small-v2	33	0.12	72.9	384
GIST-all-MiniLM-L6-v2	23	0.08	72.7	384
gte-small	33	0.12	72.3	384
bge-small-en-v1.5	33	0.12	72.2	384
MedEmbed-small-v0.1	33	0.12	71.8	384
gte-tiny	23	0.08	70.4	384
e5-small	33	0.12	68.1	384

For GenePT, we downloaded the gene text embeddings from its Zenodo repository. These embeddings were generated using OpenAI's text-embedding-ada-002 model [34], with each embedding having a dimension of 1536. This dimensionality is significantly higher than that of the embeddings produced by the SLMs.

### 2.2 Logistic regression and random forests on SLM embeddings

For each of the four two-class classification tasks, we supplied gene embeddings generated by the ten SLMs, as well as those from OpenAI, to a classifier. We measured performance using the area under the ROC curve (AUC), which ranges from 0 to 1. Higher AUC values indicate better performance.

Following the GenePT paper, we chose logistic regression and random forest as our classifiers. These are implemented using the LogisticRegression() function from the sklearn.linear_model Python package and the RandomForestClassifier() function from the sklearn.ensemble Python package, respectively. We used the default hyperparameter settings for both functions without any further tuning.

**Fig. 1:**
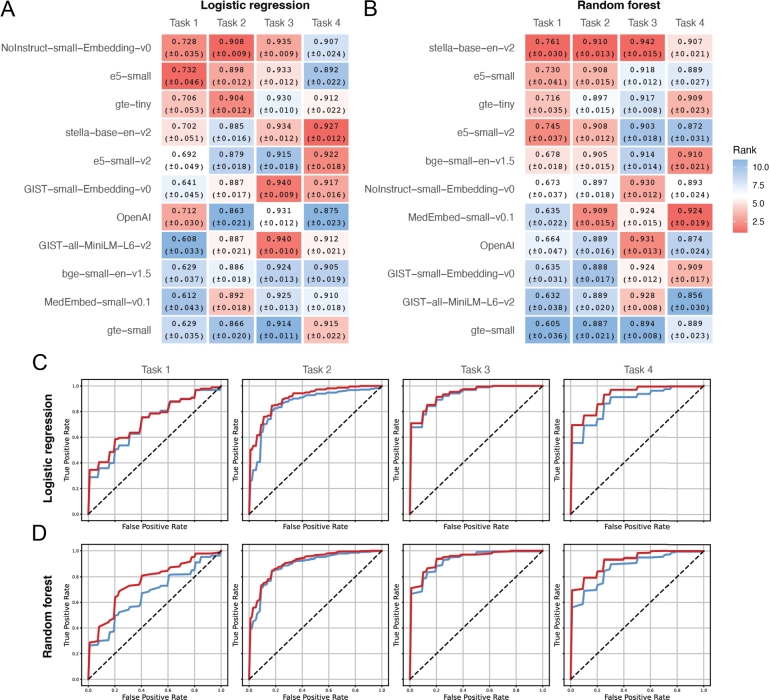
Classification performance across four tasks using sentence embeddings derived from gene text descriptions without hyperparameter tuning. **A-B.** Heatmaps showing the average ROC-AUC scores (with standard errors) for 11 embedding models evaluated on four classification tasks using **A.** logistic regression and **B.** random forest classifiers. Each cell is colored by rank (red = higher rank, blue = lower rank) across models within each task. **C-D.** ROC curves for OpenAI and the best performed embedding models: **C.** logistic regression using OpenAI (blue) and NoInstruct–small–Embedding–v0 (red); **D.** random forest using OpenAI (blue) and stella–base–en–v2 (red). OpenAI embeddings consistently underperform relative to the top SLM-based models across all tasks.

For each task, we randomly split the data into training and testing sets, comprising 90% and 10% of the total data, respectively. The classifier—either logistic regression or random forest—is trained on the training data and then tested on the test data. To minimize the randomness in the evaluation, this entire procedure (random splitting, training, and testing) is repeated 10 times. Figs. 1 A and B present the mean AUC scores from these 10 repetitions, along with standard errors. In each subplot, methods are ranked from top to bottom by their overall mean AUC across the four tasks. For each task (i.e., each column in the plot), blocks are colored based on the method's rank for that specific task among the 11 models. For example, the model e5-small achieves the highest AUC on Task 1 and is colored deep red, whereas GIST-all-MiniLM-L6-v2 has the lowest AUC on Task 1 and is colored deep blue.

Surprisingly, OpenAI's embedding does not emerge as the top performer on any of the four tasks, regardless of whether logistic regression or random forest is employed. With logistic regression, OpenAI's embedding ranks 3rd, 11th, 6th, and 11th, with an overall ranking of 7th. Moreover, two of the SLMs we evaluated, NoInstruct-small-Embedding-v0 and e5-small, consistently outperformed OpenAI's embedding across all four tasks. The AUC curves for NoInstruct-small-Embedding-v0 and OpenAI are shown in Fig. 1C, while the curves for all methods are provided in Fig. S1A.

Similar results are observed with random forest: OpenAI's embedding ranks 7th, 8.5th (mean rank shown in the case of ties), 2nd, and 9th, with an overall ranking of 8th. Additionally, a SLM, stella-base-en-v2, consistently performed better across all tasks. The AUC curves for stella-base-en-v2 and OpenAI are shown in Fig. 1D, with results for all models presented in Fig. S1B.

### 2.3 Logistic regression and random forests on SLM embeddings, with hyperparameter tuning

In the aforementioned analysis, we employed logistic regression and random forest classifiers without tuning their hyperparameters. For logistic regression, this involved using an $\ell_{2}$ penalty with a strength of 1, specified by setting penalty = ‘l2’ and C = 1.0 in the LogisticRegression() function. For random forest, we used a default configuration of 100 unpruned trees, set by n_estimators = 100 and max_depth = None in the RandomForestClassifier() function.

The sub-par performance of OpenAI's embedding observed in the previous section may be attributed to the lack of hyperparameter tuning. Therefore, in this section, we will tune these hyperparameters to see if the conclusions change. For logistic regression, we explored all combinations of penalty = [‘l1’, ‘l2’] and C = [0.01, 0.1, 1, 10, 100]. For random forest, we considered all combinations of n_estimators = [25, 50, 100, 200, 400] and max_depth = [None, 10, 20, 30].

**Fig. 2:**
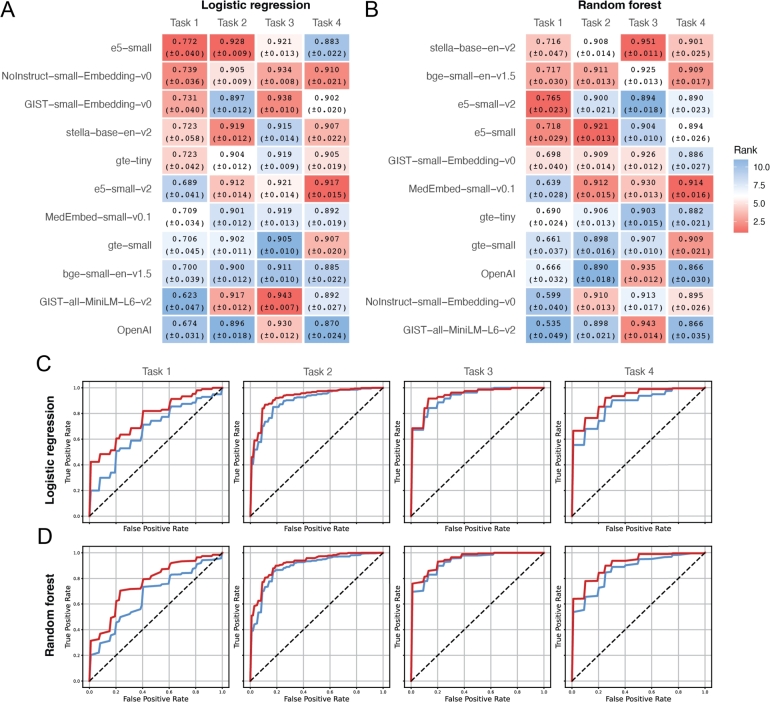
Classification performance across four tasks using sentence embeddings derived from gene text descriptions with hyperparameter tuning. **A-B.** Heatmaps showing the average ROC-AUC scores (with standard errors) for 11 embedding models evaluated on four classification tasks using **A.** logistic regression and **B.** random forest classifiers. Each cell is colored by rank (red = higher rank, blue = lower rank) across models within each task. **C-D.** ROC curves for OpenAI and the best performed embedding models: **C.** logistic regression using OpenAI (blue) and e5-small (red); **D.** random forest using OpenAI (blue) and stella–base–en–v2 (red). OpenAI embeddings consistently underperform relative to the top SLM-based models across all tasks.

Once again, we randomly divided the data for each task into training and test datasets, consisting of 90% and 10% of all samples, respectively. We employed five-fold cross-validation on the training data to determine the optimal hyperparameters. These selected hyperparameters were then used to train a classifier on the entire training dataset. The classifier was subsequently tested on the test data, which had been set aside during the hyperparameter selection and training phases. This entire process was repeated ten times to minimize variability, and the mean AUC values were calculated and reported in Figs. 2 A and B, along with standard errors. In each subplot, the methods are ordered from top to bottom according to their average AUC across the four tasks. Within each column, the color of each block reflects the model's rank on that specific task among the 11 evaluated methods.

Despite the adjustments, OpenAI's embedding still does not outperform those from the SLMs, as detailed in the figures. It fails to achieve the highest AUC value in any of the four tasks, and on average across these tasks, it is ranked 11th and 9th out of 11 embeddings when using logistic regression and random forest, respectively. This means that for each task, at least one SLM embedding performs better than OpenAI's. On average, all ten SLMs outperform OpenAI when using logistic regression, and eight do so when using random forest. Fig. 2C shows the AUC curves for e5-small (the top performer) and OpenAI, with results for all models under logistic regression shown in Fig. S2A. Similarly, Fig. 2D displays the AUC curves for stella-base-en-v2 (the top performer) and OpenAI, with the corresponding results for all models under random forest presented in Fig. S2B.

Comparing Fig. 2 A with Fig. 1 A, we observe that hyperparameter tuning enhances the AUC values for nine out of ten SLMs using logistic regression, with an average improvement of 0.0099. In contrast, when using random forest, as shown by comparing Fig. 2 B with Fig. 1 B, tuning improves AUC values for only four SLMs, with an average decrease of 0.0002. This suggests that tuning the hyperparameters of the classifiers does not consistently enhance performance. This phenomenon is likely due to the small sample sizes of our tasks, coupled with high-dimensional input (the embeddings). For instance, in tasks 1 and 4, there are fewer than 50 samples in the minor class. This limited sample size, along with the high dimensionality of the input embeddings, contributes to significant randomness in the selection of optimal hyperparameters and high variance in the test AUC, especially for more complicated methods like random forest.

### 2.4 SLM embeddings with fine-tuning

So far, we have demonstrated that the embeddings produced by SLMs often perform as well as, or even better than, those generated by OpenAI in our four tasks. These unexpected results render our initial plan to fine-tune SLMs to catch up with OpenAI's performance unnecessary. However, we still wish to explore the fine-tuning of these SLMs in this section, with the aim of determining whether fine-tuning can further enhance their performance and thereby extend their advantage over OpenAI.

This fine-tuning approach differs significantly from the methods considered in previous sessions. Previously, all methods involved two independent steps. Initially, a language model (either OpenAI's or a SLM) would generate an embedding from text. Subsequently, a classifier (logistic regression or random forest) would use this embedding to predict the class label. These two steps were independent, meaning the embeddings generated in the first step did not change during the classifier's training. Conversely, the fine-tuning approach employs a single integrated model. It involves adding a fully connected layer to the language model, effectively transforming the language model from an embedding generator into a classification model. This single deep-neural-network-based model is trained by taking NCBI gene descriptions as input and outputting the predicted class labels directly. During training, both the weights in the language model and in the final layer are adjusted. Thus, the embeddings from the language model are tuned specifically for the classification task, potentially enhancing classification accuracy.

For fine-tuning, we randomly divided the data for each task into training, evaluation, and test sets in proportions of 80%, 10%, and 10%, respectively. The fine-tuning process consisted of two stages. In the first stage, the model was trained on the training data and evaluated on the evaluation data to determine the optimal training hyperparameters. In the second stage, the model, configured with the best hyperparameters, was further trained on a combined set of the training and evaluation datasets, which accounted for 90% of all data, for several additional epochs to maximize the use of available information. Finally, this model was applied to the test data, which had been isolated from all fine-tuning and training activities, to compute the AUC score. This entire procedure was repeated ten times, and the mean AUC values were reported.

In the first stage of fine-tuning, we initialized the model with the following parameters: learning_rate = 1e-5, num_train_epochs = 20, max_grad_norm = 0.7, warmup_ratio = 0.1, and weight_decay = 0.2, with other parameters set to their default values. To prevent overfitting, we set up the early stopping (EarlyStoppingCallback(early_stopping_patience = 5)) and the ReduceLROnPlateau scheduler (factor = 0.8, patience = 2) to gradually decrease the learning rate. In the second stage, the parameters were adjusted to learning_rate = 1e-6, num_train_epochs = 10, max_grad_norm = 0.7, and weight_decay =0.2, with other settings remaining as default.

The computation was conducted on a single NVIDIA TITAN Xp GPU, an older model released in April 2017. The total time spent on the entire fine-tuning process, including data loading, model loading, and both stages of fine-tuning, is detailed in Table 3. For the two models with 23 million parameters, fine-tuning all four tasks takes about 20 minutes. Models with 33 million parameters take no more than 40 minutes, while the largest model, stella-base-en-v2, which has 55 million parameters, requires approximately one and a half hours. This demonstrates that the computational load is generally quite manageable, even on dated hardware.

**Table 3:** Computational time (in minutes) spent on fine-tuning SLMs under each task.

Models	**Table:** Time on Task 1	**Table:** Time on Task 2	**Table:** Time on Task 3	**Table:** Time on Task 4	**Table:** Mean Time	**Table:** Rank of Mean Time
GIST-small-Embedding-v0	33	36	32	30	32.8	6
NoInstruct-small-Embedding-v0	26	33	26	24	27.3	3
stella-base-en-v2	87	94	82	79	85.5	10
e5-small-v2	32	35	31	32	32.5	5
GIST-all-MiniLM-L6-v2	18	23	19	18	19.5	1
gte-small	36	43	37	32	37.0	8
bge-small-en-v1.5	30	38	34	31	33.3	7
MedEmbed-small-v0.1	37	44	37	39	39.3	9
gte-tiny	19	24	21	17	20.3	2
e5-small	26	31	29	25	27.8	4

**Fig. 3:**
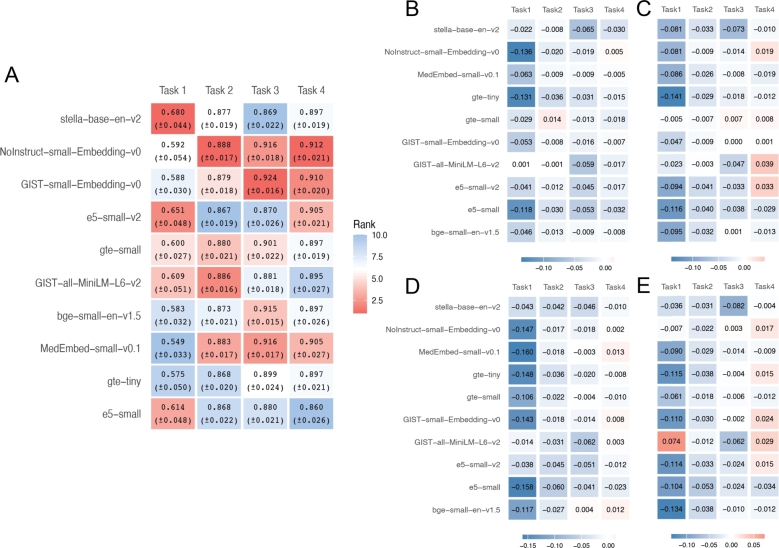
Fine-tuning performance across four classification tasks using gene text embeddings. **A.** Average ROC-AUC scores (with standard errors) across four gene classification tasks by fine-tuning 11 different SLMs. Each cell is colored by the rank of performance within each task (red = higher rank, blue = lower rank). **B-E.** Differences in average ROC-AUC between the fine-tuning setting (panel A) and the settings shown in Fig. 1, Fig. 2. **B.** Differences for logistic regression with default hyperparameters vs. fine-tuned SLMs (A – Fig. 1A). **C.** Differences for random forest with default hyperparameters vs. fine-tuned SLMs (A – Fig. 1B). **D.** Differences for logistic regression with hyperparameter tuning vs. fine-tuned SLMs (A – Fig. 2A). **E.** Differences for random forest with hyperparameter tuning vs. fine-tuned SLMs (A – Fig. 2B).

Fig. 3A shows the average AUC values from ten repetitions of fine-tuning the ten SLMs, with the corresponding AUC curves provided in Fig. S3. Figs. 3B,C and 3D,E display the performance improvements relative to the results in Figs. 1 (i.e., without hyperparameter tuning) and 2 (i.e., with hyperparameter tuning), respectively. Unexpectedly, across all comparisons—whether using logistic regression or random forest, and whether or not hyperparameter tuning is applied—fine-tuning generally does not yield noticeable improvements in classification accuracy, if any. It is worth noting that we have tried more settings of training parameters than what we have described above. Specifically, we have tried learning_rate = [1e-6, 5e-6, 1e-5, 5e-5, 1e-4], weight_decay = [0.0, 0.1, 0.2, 0.3, 0.4], warmup_ratio = [0.00, 0.05, 0.10, 0.15, 0.20], max_ grad_norm = [0.6, 0.7, 0.8, 0.9, 1.0], and num_train_ epochs = [5, 10, 15, 20, 25], and have not obtained results that are significantly better than those presented in Fig. 3.

### 2.5 Further investigations

In this section, we present additional computational experiments aimed at exploring potential reasons for the observed outperformance of SLMs, as well as other factors that may influence our findings.

**Fig. 4:**
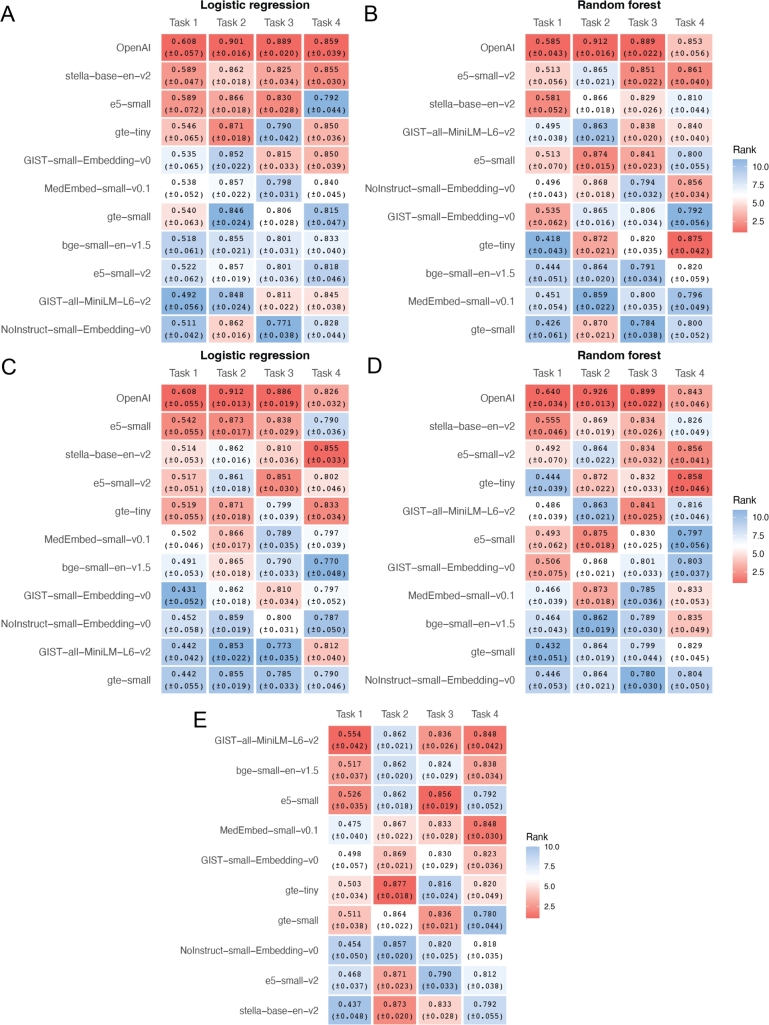
Classification performance across four tasks using sentence embeddings derived from gene names alone without hyperparameter tuning (**A-B.**), with hyperparameter tuning (**C-D.**), and by fine-tuning (**E.**).

First, we conducted a sensitivity analysis to assess how model performance varies with the amount of input information. In the main analysis, the input to the language models included both gene symbols and their corresponding NCBI summaries. Here, we repeated the entire analysis using only gene symbols as input—this includes all settings: logistic regression and random forest, both with and without hyperparameter tuning, as well as SLM fine-tuning. The average AUC values across 10 repetitions are shown in Fig. 4, and the corresponding AUC curves are provided in Figs. S4-S6. As expected, we observed a general decrease in mean AUC scores across all models when using only gene symbols, reflecting the reduced information content in the input. Notably, under this setup, OpenAI's embeddings consistently outperformed those from all SLMs across all configurations, regardless of classifier type or whether hyperparameter tuning was applied. This finding suggests that OpenAI's embedding model possesses the most extensive “knowledge” about human genes, likely acquired during pretraining. However, this advantage disappears once the NCBI gene descriptions are included as input, as demonstrated in the previous sections.

**Fig. 5:**
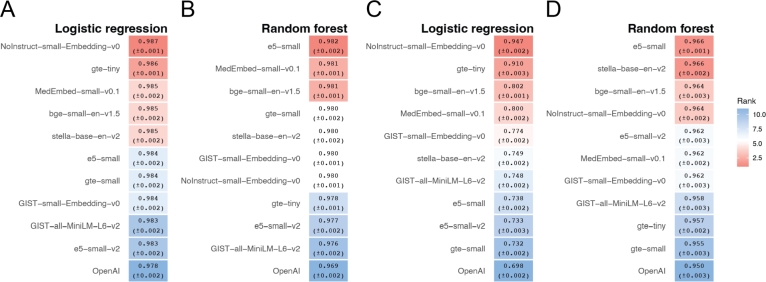
Performance of text embeddings on a large multi-class classification task without (**A-B.**) and with (**C-D.**) hyperparameter tuning.

All four classification tasks we previously considered had limited sample sizes. To explore how our conclusions might change with a larger dataset, we analyzed an additional task introduced in the GenePT paper, which involves classifying 28,620 genes into 15 common functional categories. These class labels were curated as part of the resource described in [10]. Notably, the dataset is highly imbalanced, with over 20,000 genes labeled as protein-coding—far outweighing the other classes. Fig. 5 (and Fig. S7) presents the classification performance of 11 models under both default and tuned hyperparameter settings across two classifiers. Interestingly, OpenAI embeddings consistently underperformed relative to those from SLMs, ranking last in all panels of Fig. 5A–D. We also observed, once again, that hyperparameter tuning did not lead to consistent improvements; in fact, for logistic regression, tuning noticeably reduced performance (Fig. 5C). This counterintuitive result may be due to overfitting introduced by the aggressive grid search of regularization hyperparameters, or by interactions between the chosen regularization strength and the data imbalance. In contrast, all models achieved strong performance under default hyperparameter settings, with average AUC scores exceeding 0.95 and low standard errors. Overall, our findings on this larger dataset are consistent with those from the smaller datasets.

The SLMs evaluated in our study were not specifically pre-trained on molecular biology corpora. (Note that MedEmbed-small-v0.1 is not an exception: although it was fine-tuned on clinical note–derived synthetic triplets, these are primarily patient-facing or oriented toward clinical trials. It is still considered a general-purpose model and performs well on the MTEB benchmark.) Given the molecular-biology focus of our tasks, we sought to examine whether a domain-specific model might yield improved performance. To this end, we included BioBERT (version: BioBERT-base-cased-v1.1) [35], a BERT-based language model pre-trained on PubMed abstracts and PMC full-text articles, which is designed to capture the specialized vocabulary and linguistic structure of biomedical literature. We repeated all prior analyses using BioBERT, including classification with logistic regression and random forest (with and without hyperparameter tuning), using embeddings generated from either both NCBI gene summaries and gene symbols or from gene symbols alone, as well as SLM fine-tuning. The results are summarized in the Supplementary Table 1 (Supplementary_Table_1.xlsx). When using gene symbol embeddings, BioBERT consistently ranked among the top seven models; when using full gene descriptions, it occasionally achieved top-tier performance. However, its performance in fine-tuning tasks was inconsistent—ranking highly in some cases but poorly in others. Overall, these findings do not suggest a clear or consistent advantage of BioBERT over the general-purpose SLMs.

**Fig. 6:**
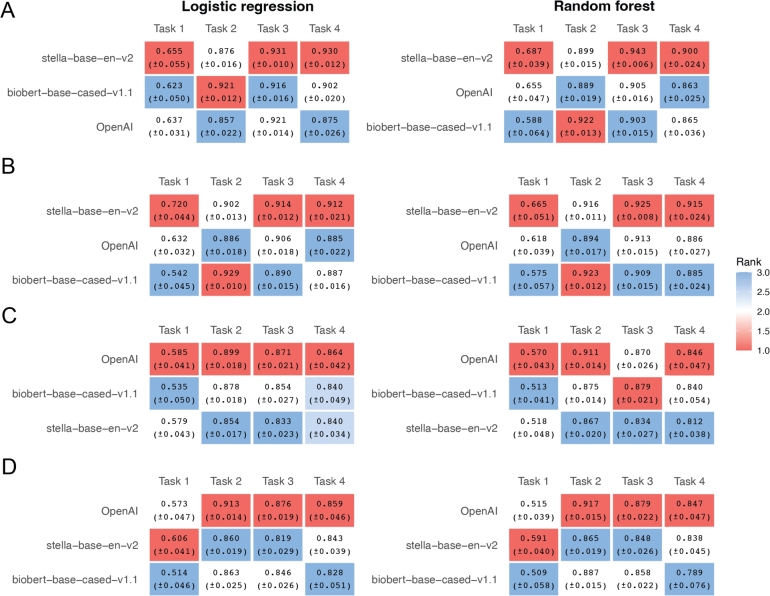
Effect of dimensionality truncation on model performance across four classification tasks. This figure compares the performance of three embedding models—OpenAI (1536 → 384), stella-base-en-v2 (768 → 384), and biobert-base-cased-v1.1 (768 → 384)—after truncating their embeddings to 384 dimensions. Panels **A** and **B** use text embeddings, while **C** and **D** use name embeddings. Panels **A** and **C** show results without hyperparameter tuning, and panels **B** and **D** include hyperparameter tuning.

One possible explanation for the lower performance of the larger OpenAI model in our setting is the high dimensionality of its embeddings, which may pose a disadvantage given the small sample sizes and class imbalance in our datasets. To assess this possibility, we truncated the embeddings generated by OpenAI (original dimension: 1536), stella-base-en-v2 (768), and BioBERT-base-cased-v1.1 (768) to 384 dimensions—the embedding length of the other SLMs evaluated in this study. Comparing the results in Fig. 6 (with AUC curves shown in Figs. S8–S9) to those in Figs. 1, 2, and 4 A-D, we found that dimensionality reduction does not improve OpenAI's performance and may even slightly degrade it. These results suggest that embedding dimensionality alone does not explain the model's underperformance; even at a reduced dimensionality, OpenAI's embeddings fail to outperform those from smaller SLMs.

For completeness, in addition to reporting AUC scores, we also provide supplementary evaluation metrics—including precision (Figs. S10–S17), recall (Figs. S18–S25), and F1 score (Figs. S26–S33)—for all analyses described above. Despite differences in the specific metrics, the overall trends observed with AUC are largely consistent across these additional evaluations.

## 3 Conclusions and discussion

Recently, LLMs have been developed and utilized for describing and analyzing gene-expression data. While training a gene-expression foundation model can be labor-intensive and computationally prohibitive for most labs, GenePT's approach of using gene embeddings generated by OpenAI's text-embedding tools offers an affordable and efficient alternative. However, privacy concerns and other potential issues associated with the closed-source, online service nature of OpenAI's embeddings may hinder the widespread adoption of GenePT's approach. In this paper, we have explored the possibility of using open-source SLMs, which can be readily installed and run locally, as alternatives to OpenAI's text-embedding. Our evaluation has focused on the four gene-property prediction tasks considered in the GenePT paper.

While the expectation was that the embeddings from these SLMs could not compete with those from OpenAI, and that fine-tuning these SLMs might be necessary, the observations have been surprisingly different. First, we have found that even without fine-tuning, there are SLMs that outperform OpenAI on every task. Some SLMs even surpass OpenAI across all four tasks. This holds true regardless of whether logistic regression or random forest is used, and irrespective of whether the hyperparameters in the classifier are carefully tuned. This suggests that SLMs can be a legitimate alternative to OpenAI's embedding tool for applications similar to those we have considered. Our observation aligns with findings from recent studies showing that light language models can rival or surpass larger models in targeted or domain-specific tasks [36], [37], [38]. While the precise reasons for OpenAI's underperformance are difficult to isolate, our results in the “Further investigations” section suggest that a combination of factors—including pretraining data, input type, and task structure—contribute to the superior performance of smaller models in this context.

Second, we have found that although fine-tuning SLMs can be readily performed, it did not result in enhanced performance. This outcome is not entirely surprising. In fact, previous literature in more general settings of large language models, unrelated to genetics, has reported that fine-tuning pretrained language models is often brittle [39] and prone to degraded performance under small training sample sizes [40]. It is also well documented that fine-tuning can induce catastrophic forgetting (see, e.g., [41], [42], [43], [44]), which significantly reduces model generalizability—not only across tasks, but also from training to test data. The limited effectiveness of fine-tuning in our experiments likely reflects both general challenges of fine-tuning (e.g., sensitivity, instability, and forgetting) and the specific characteristics of our data (small size, high imbalance, short input sequences). While our research does not preclude the possibility that fine-tuning might improve the performance of SLMs for gene analysis, and further investigation into this matter is certainly valuable, our findings suggest that simple, straightforward fine-tuning of SLMs may not consistently enhance their performance for gene analysis with small sample sizes.

Third, we have discovered that hyperparameter tuning slightly improves the performance of logistic regression but does not enhance the performance of random forest. Thus, using the default settings of these classifiers in Python functions can be a practical and reliable choice. Additionally, when comparing the AUC values generated by logistic regression with those produced by random forest, we observe that random forest often does not outperform logistic regression, despite its higher complexity and capability to capture non-linear relationships between features. These somewhat counterintuitive findings may be attributed to a couple of factors. First, the datasets we analyzed are characterized by high dimensions and small sample sizes, which makes reliable model selection very challenging. Second, the correlations or associations between the dimensions of the embeddings remain an open area of research, and it is still unclear whether random forest can effectively capture such relationships. Overall, using gene-description embeddings for gene analysis is a recent development. Further advancements in this field may hinge on a deeper understanding of these high-dimensional embeddings and how to utilize them most effectively. Additionally, exploring effective dimensionality reduction of these high-dimensional embeddings could also be a meaningful direction for future research.

Our current work has several limitations. First, its scope is limited to gene-level analysis, with a particular focus on gene-property prediction tasks. GenePT also has potential applications at the cell level, such as cell-type clustering. As highlighted by [45], the question of how to efficiently combine gene embeddings with gene expression data to enhance the performance of gene-level tasks remains largely unanswered. Extending our analysis to cell-level tasks represents a challenging but potentially valuable future direction. Second, the effectiveness of our approach may depend on the quality and completeness of the text summaries available for the biological entities. In practice, such summaries can be incomplete, inconsistent, or entirely absent—especially for poorly characterized entities or those from non-human species—thereby limiting the generalizability of our findings. Third, several of the classification tasks we examined suffer from small sample sizes and class imbalance. These constraints reduce statistical power, increase variability in performance estimates, and complicate fine-tuning and hyperparameter selection. While we employed repeated sampling and cross-validation to mitigate these issues, the inherent limitations of small datasets persist. Future work involving larger, more diverse datasets and additional biological tasks will be critical for validating and expanding the applicability of embedding-based classification frameworks.

## Code availability

The source code is available at https://github.com/RavenGan/FinetuneEmbed.

## CRediT authorship contribution statement

**Dailin Gan:** Writing – review & editing, Writing – original draft, Visualization, Validation, Software, Methodology, Investigation, Formal analysis, Data curation, Conceptualization. **Jun Li:** Writing – review & editing, Writing – original draft, Supervision, Resources, Project administration, Methodology, Funding acquisition, Conceptualization.

## Declaration of Competing Interest

The authors have no conflicts of interest to declare.

## Data Availability

All datasets used in the study have been published and cited in the main body.

## Appendix A Supplementary material

MMC 1 Supplementary figures. MMC 1

MMC 2 Supplementary Table 1. MMC 2
