# Supplementary material for: Small, open-source text-embedding models as substitutes to OpenAI models for gene analysis
Source: Comput Struct Biotechnol J. 2025 Aug 6;27:3598–608. doi: 10.1016/j.csbj.2025.07.053 (PMC12359258; doi:10.1016/j.csbj.2025.07.053)
Supplement: MMC 1 — Supplementary figures. [file mmc1.pdf]

Supplementary materials

Dailin Gan[1] and Jun Li[1*]

[1]*Department of Applied and Computational Mathematics and Statistics, University of Notre Dame, Notre Dame, IN, USA*
[*]*To whom correspondence should be addressed. Tel: +1 574 631 3429; Fax: +1 574 631 4822; Email: jun.li@nd.edu*

Supplementary Figures

ROC curves

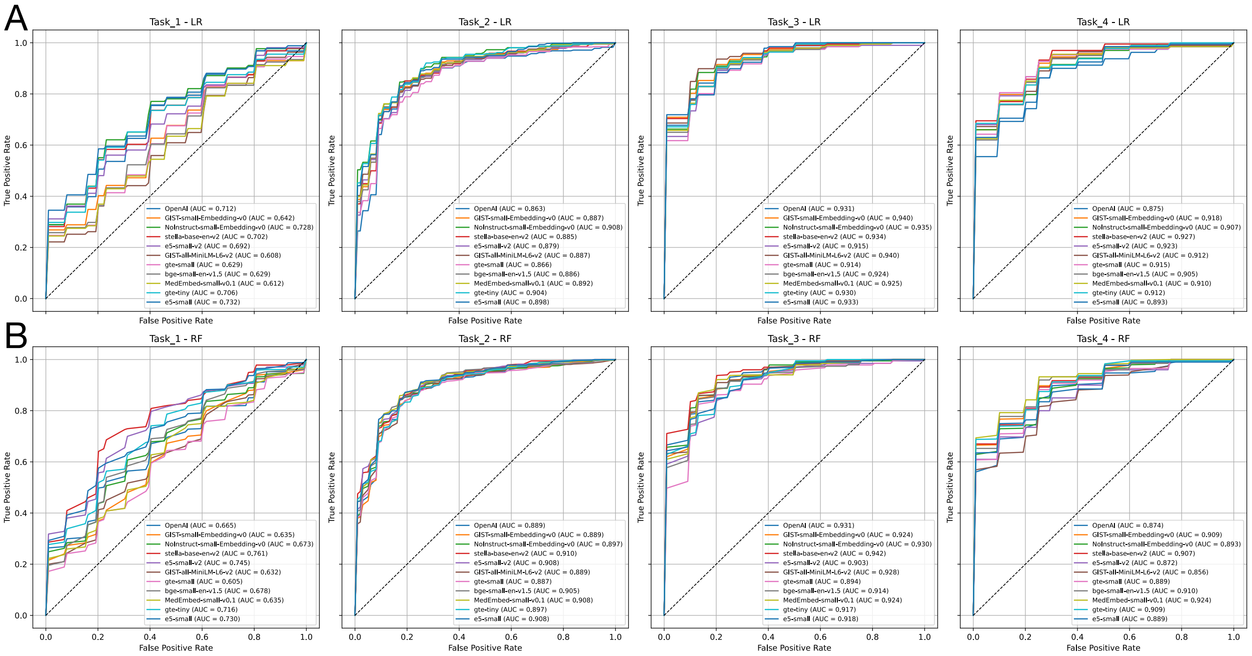

Figure S1: ROC curves for text embeddings across four classification tasks using logistic regression and random forest without hyperparameter tuning. Receiver operating characteristic (ROC) curves are shown for 11 sentence-level language models using gene text embeddings across four biomedical classification tasks. Panel A displays results using logistic regression (LR), and Panel B shows results using random forest (RF). Each curve represents the average performance of one embedding model, with the corresponding AUC score reported in the legend.

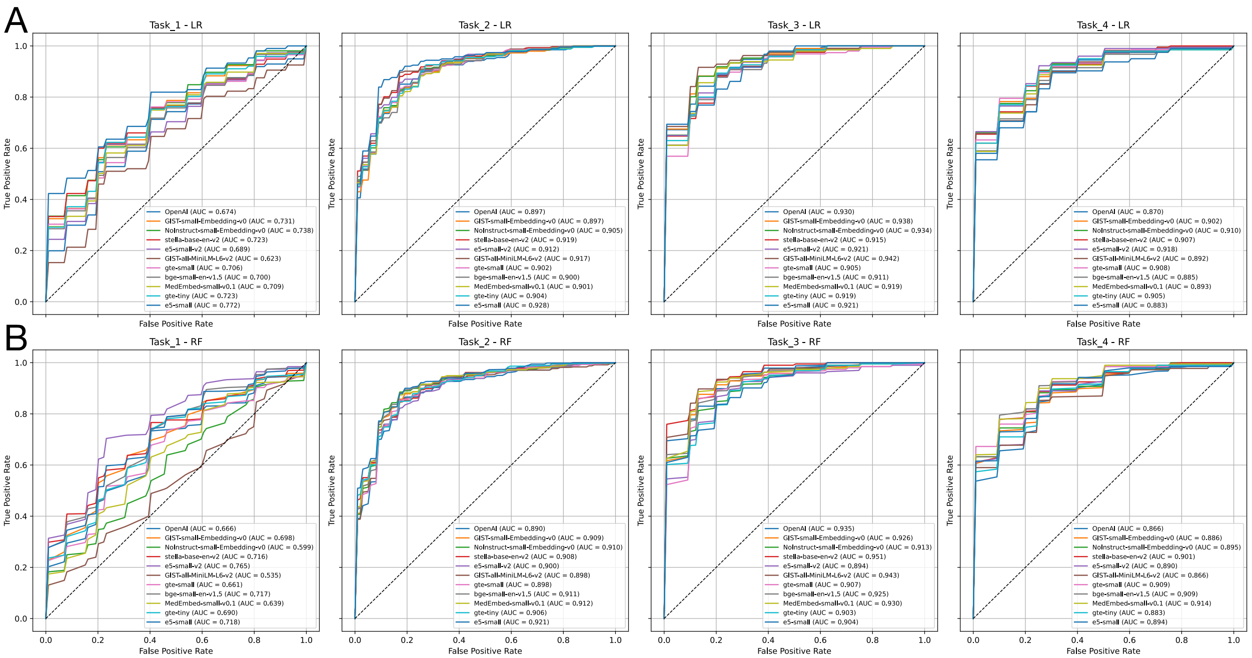

Figure S2: ROC curves for text embeddings across four classification tasks using logistic regression and random forest with hyperparameter tuning. Receiver operating characteristic (ROC) curves are shown for 11 sentence-level language models using gene text embeddings across four biomedical classification tasks. Panel A displays results using logistic regression (LR), and Panel B shows results using random forest (RF). Each curve represents the average performance of one embedding model, with the corresponding AUC score reported in the legend.

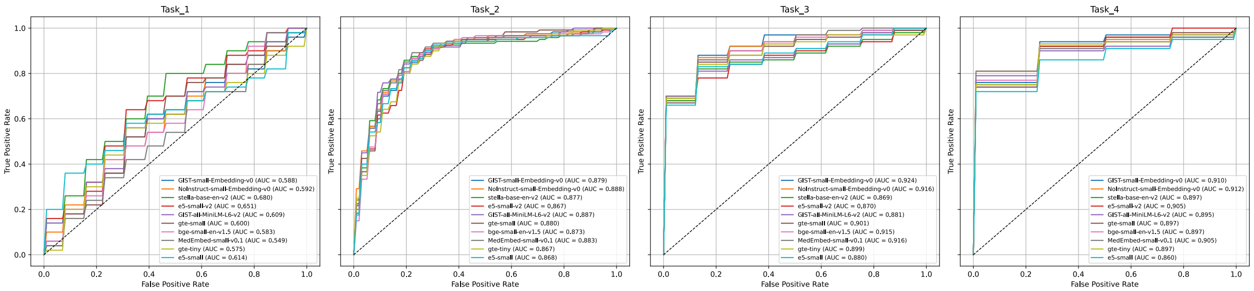

Figure S3: ROC curves for fine-tuned models across four classification tasks. Receiver operating characteristic (ROC) curves are shown for 10 SLMs fine-tuned. Each curve represents a model's performance, with corresponding AUC values reported in the legend.

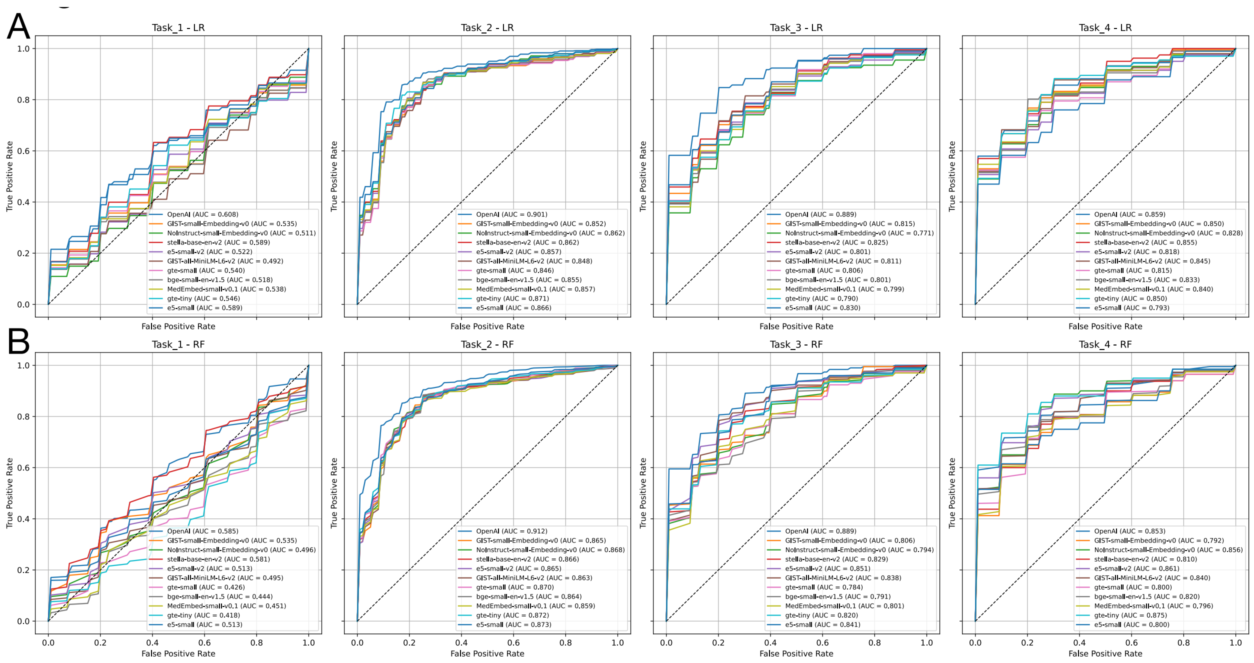

Figure S4: ROC curves for embeddings generated using gene symbols only, evaluated across four classification tasks with logistic regression and random forest, without hyperparameter tuning. Receiver operating characteristic (ROC) curves are shown for 11 language models across four classification tasks. Panel A displays results using logistic regression (LR), and Panel B shows results using random forest (RF). Each curve represents the average performance of one embedding model, with the corresponding AUC score reported in the legend.

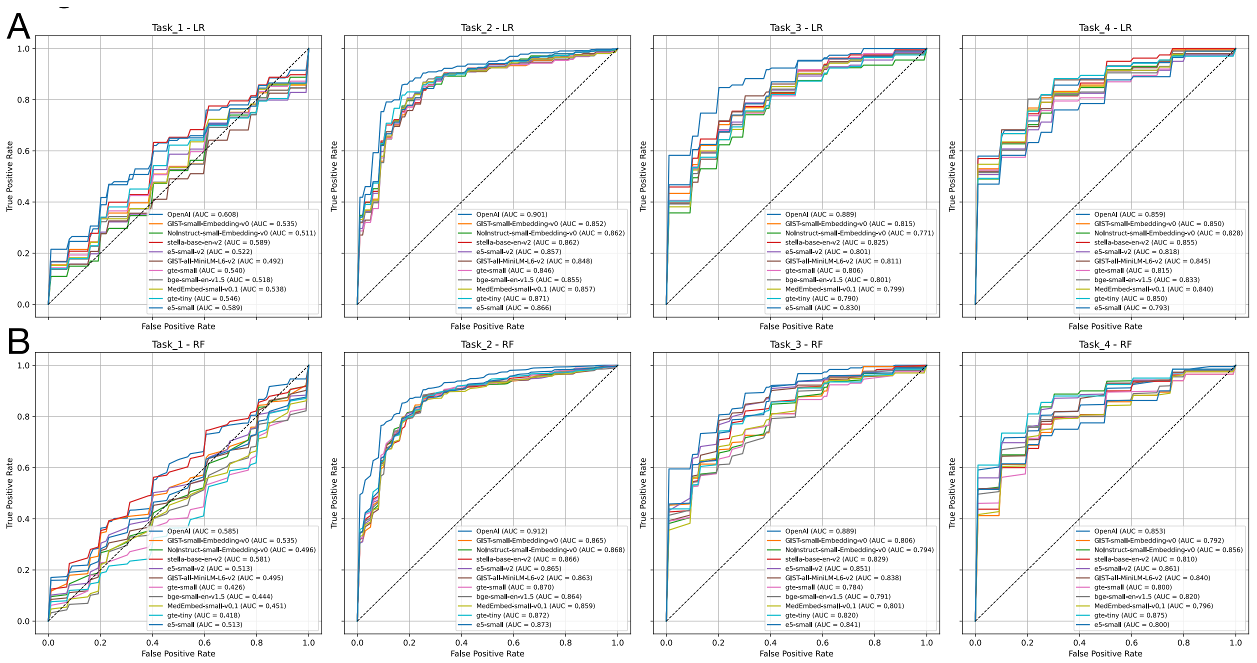

Figure S5: ROC curves for embeddings generated using gene symbols only, evaluated across four classification tasks with logistic regression and random forest, with hyperparameter tuning. Receiver operating characteristic (ROC) curves are shown for 11 language models across four classification tasks. Panel A displays results using logistic regression (LR), and Panel B shows results using random forest (RF). Each curve represents the average performance of one embedding model, with the corresponding AUC score reported in the legend.

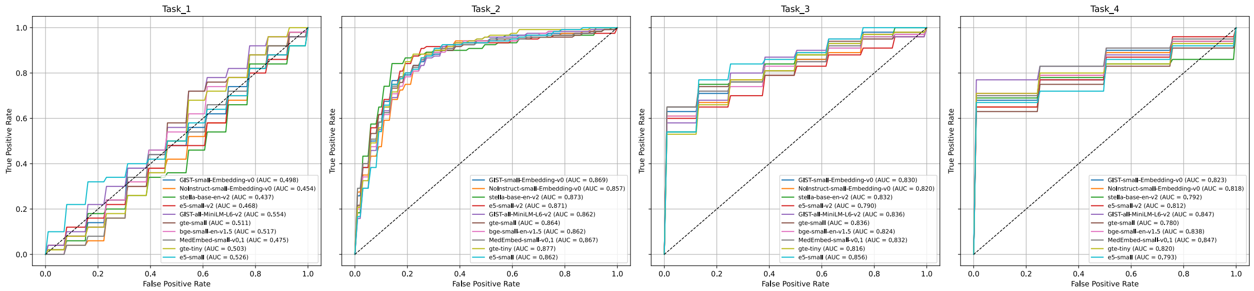

Figure S6: ROC curves for fine-tuned models using gene symbols only, evaluated across four classification tasks. Receiver operating characteristic (ROC) curves are shown for 10 SLMs. Each curve represents a model's performance, with corresponding AUC values reported in the legend.

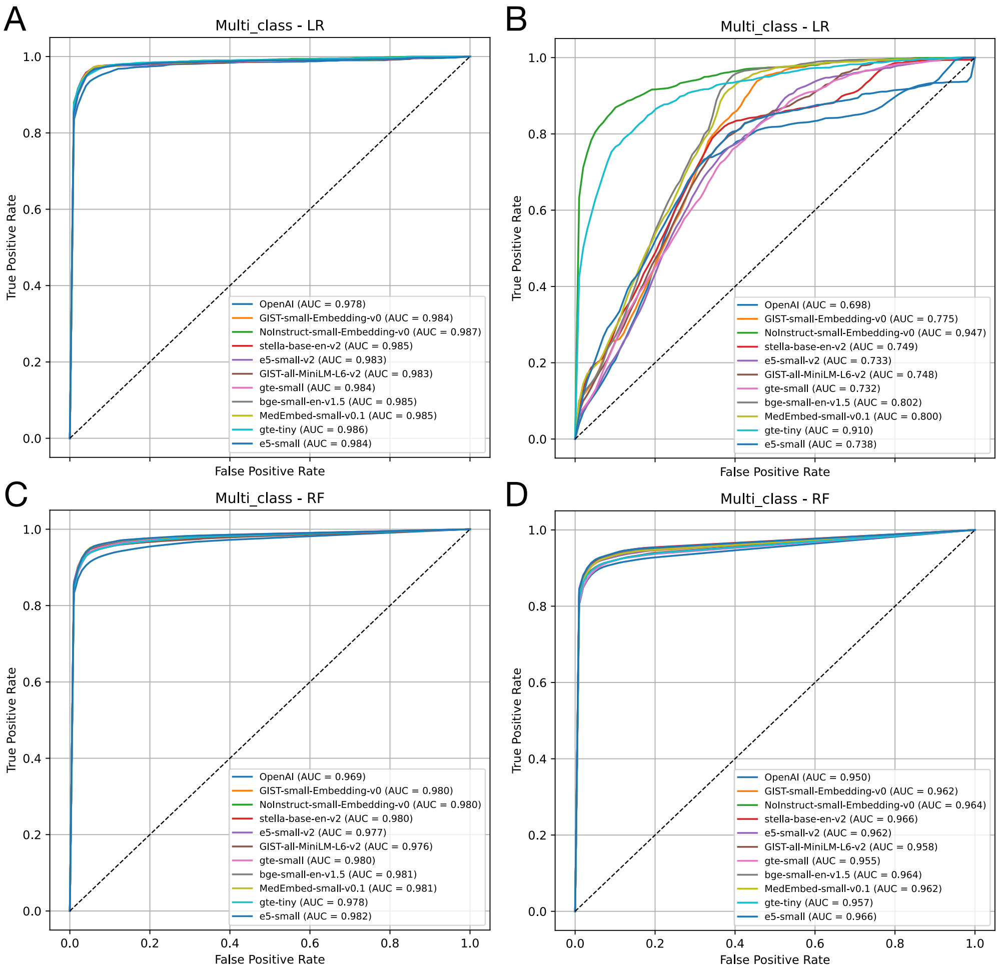

Figure S7: ROC curves for text embeddings on a large multi-class classification task (15 classes). Receiver operating characteristic (ROC) curves are shown for 11 language models evaluated data that consists of 28,620 samples across 15 functional gene classes. Models were assessed using logistic regression (LR; Panels A and B) and random forest (RF; Panels C and D). (A) and (C) display results without hyperparameter tuning. (B) and (D) show results with hyperparameter tuning. Each curve represents a model's performance, with the corresponding macro-averaged AUC score reported in the legend.

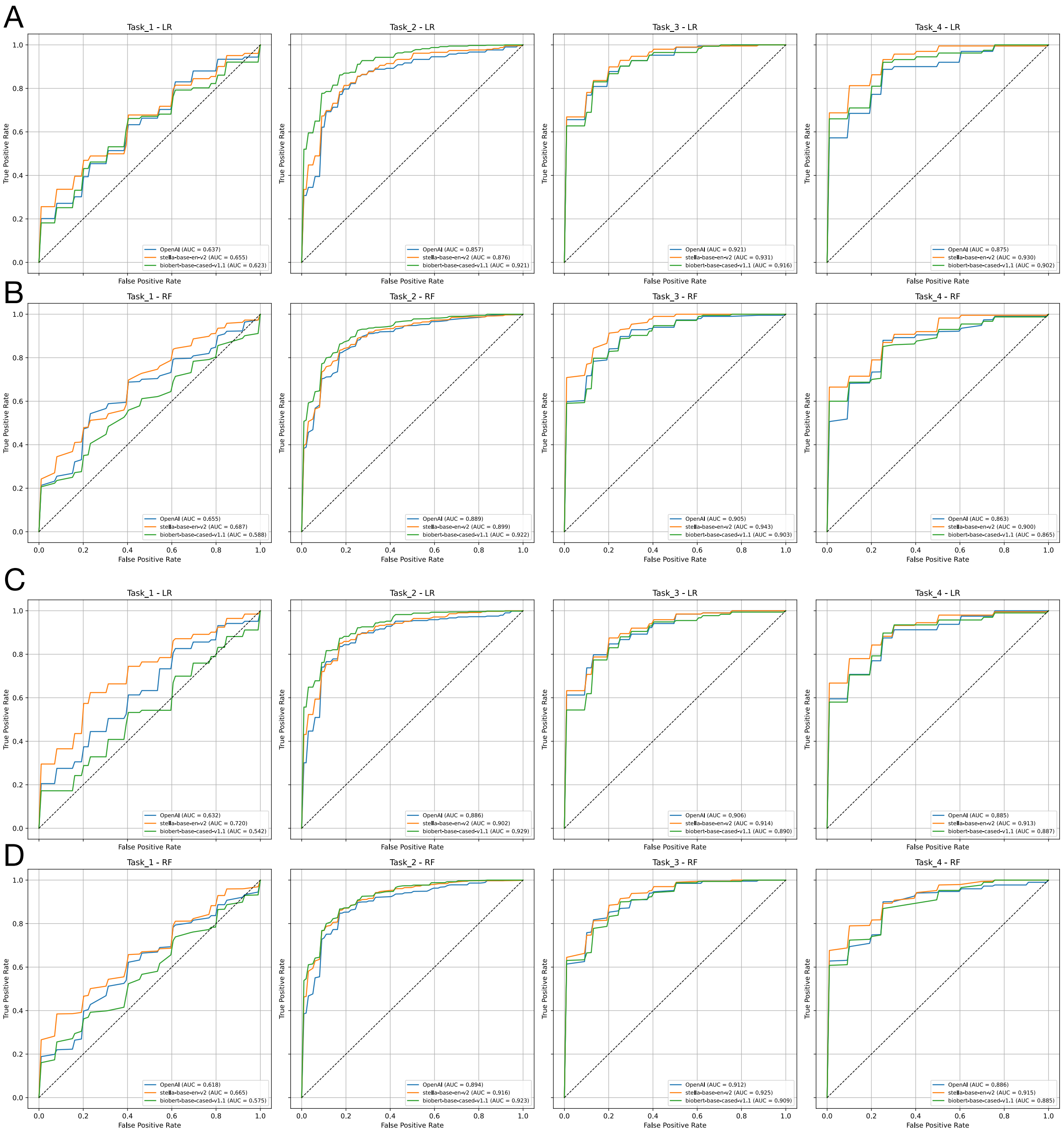

Figure S8: Comparison of ROC curves for text embedding under truncation settings with and without hyperparameter tuning. Panels A and B show results without hyperparameter tuning for logistic regression (LR) and random forest (RF), respectively. Panels C and D present results with hyperparameter tuning for LR and RF, respectively. Each subpanel corresponds to a different classification task.

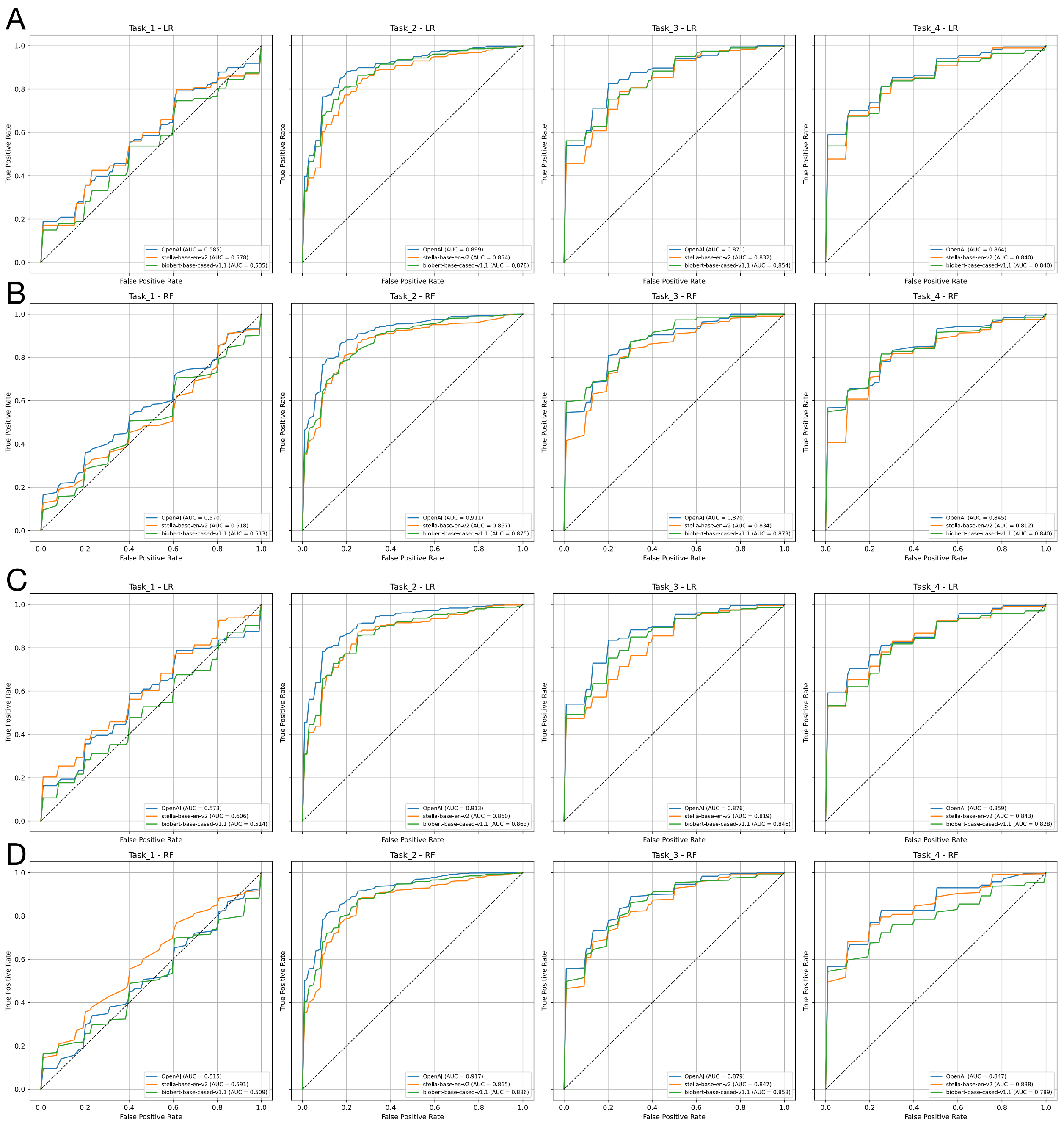

Figure S9: Comparison of ROC curves for gene embedding generated using gene names alone under truncation settings with and without hyperparameter tuning. Panels A and B show results without hyperparameter tuning for logistic regression (LR) and random forest (RF), respectively. Panels C and D present results with hyperparameter tuning for LR and RF, respectively. Each subpanel corresponds to a different classification task.

Precision

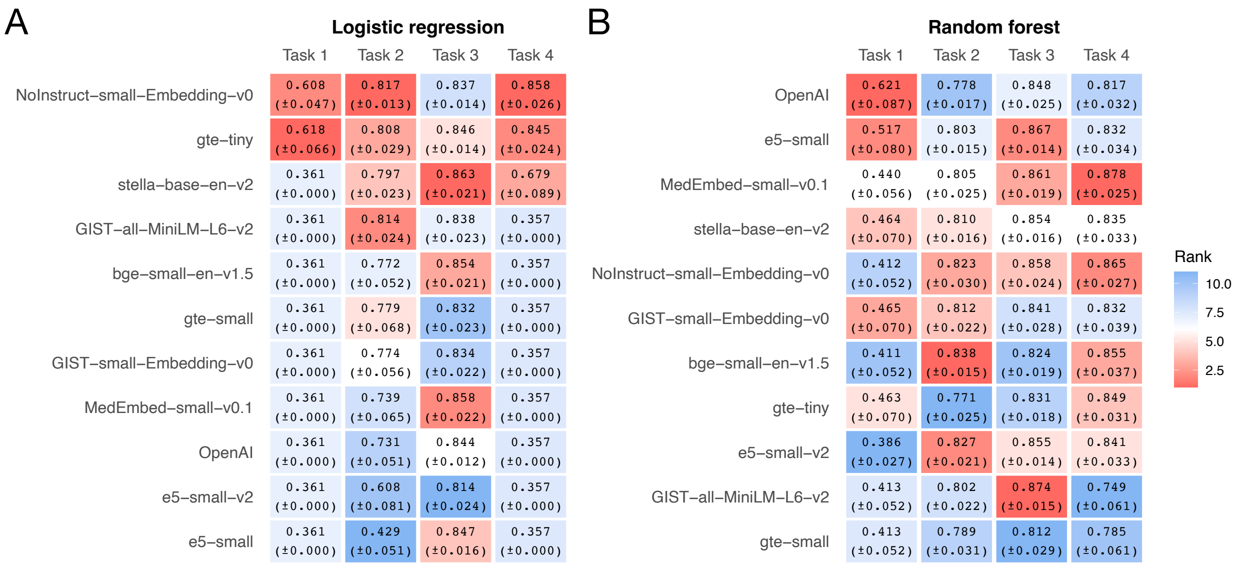

Figure S10: Classification performance across four tasks using gene embeddings derived from gene text descriptions without hyperparameter tuning.

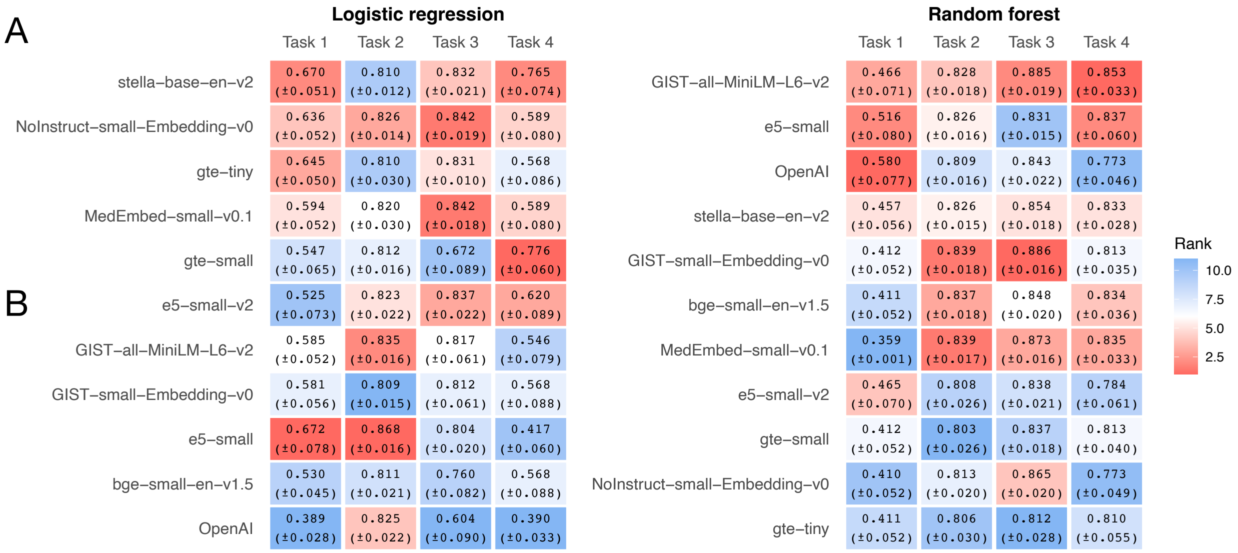

Figure S11: Classification performance across four tasks using gene embeddings derived from gene text descriptions with hyperparameter tuning

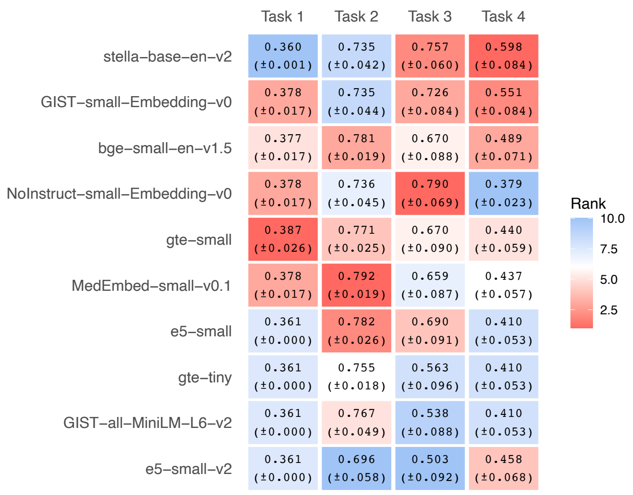

Figure S12: Fine-tuning performance across four classification tasks using gene text embeddings.

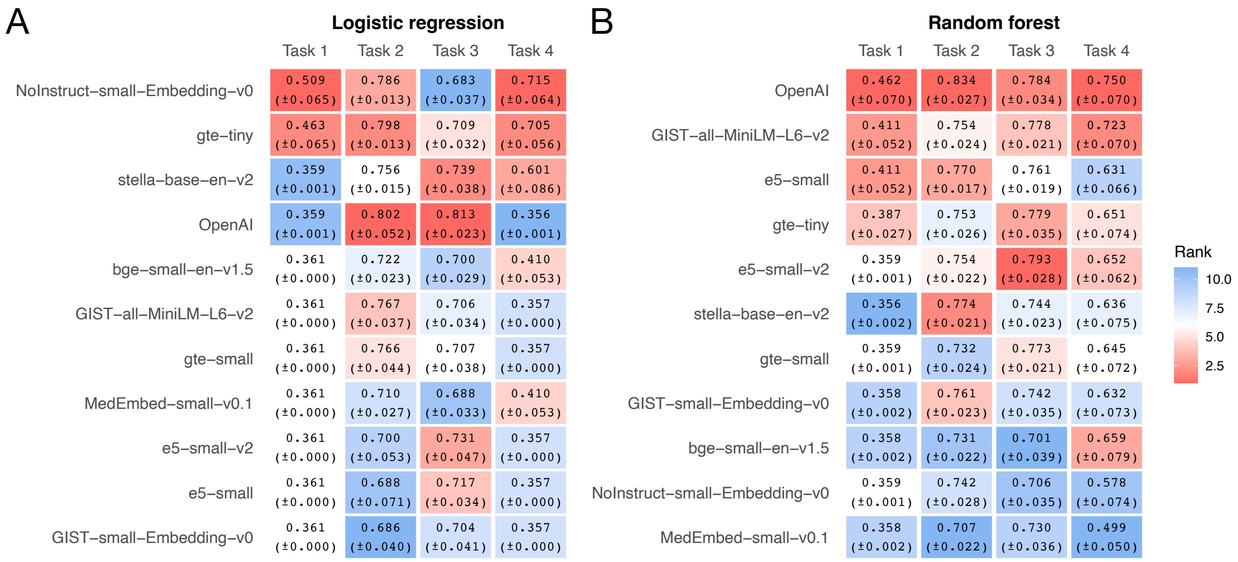

Figure S13: Classification performance across four tasks using gene embeddings derived from gene symbols alone without hyperparameter tuning.

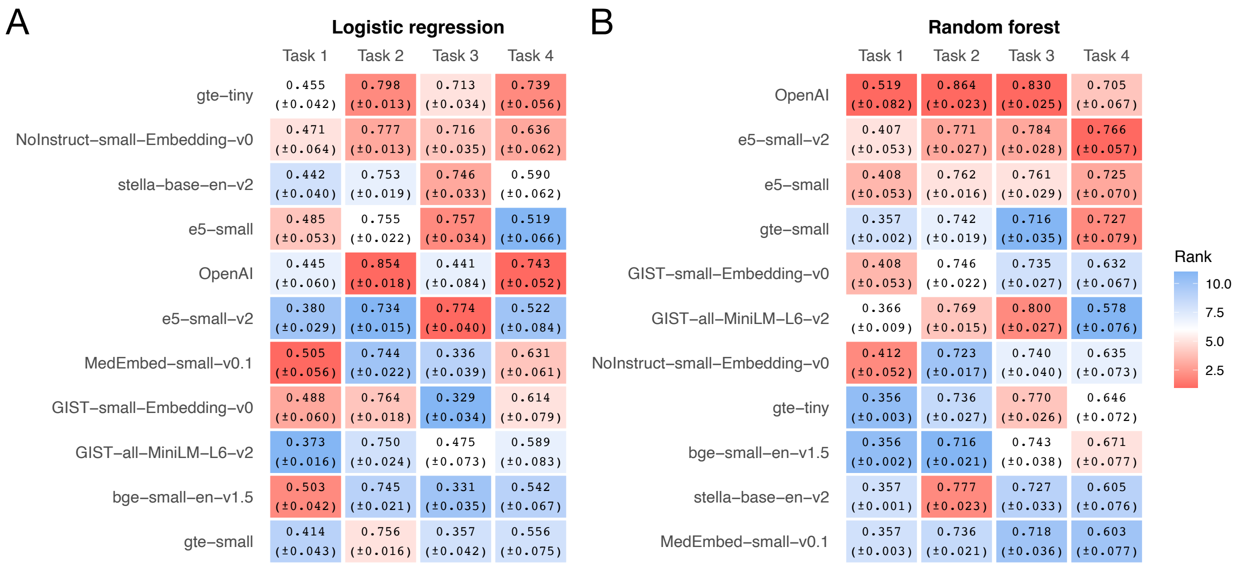

Figure S14: Classification performance across four tasks using gene embeddings derived from gene symbols alone with hyperparameter tuning.

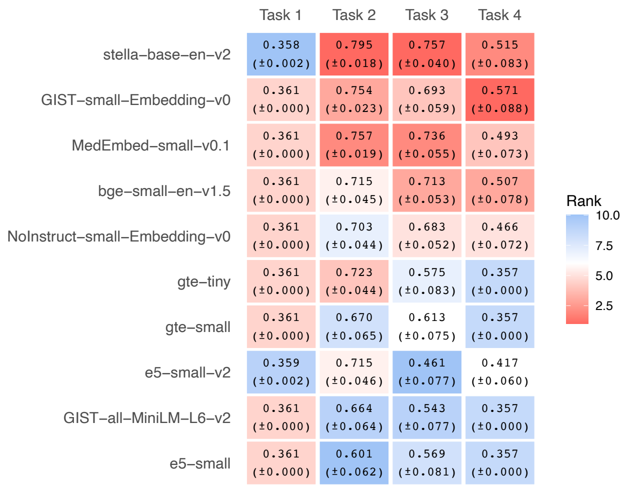

Figure S15: Fine-tuning performance across four classification tasks using gene symbol embeddings.

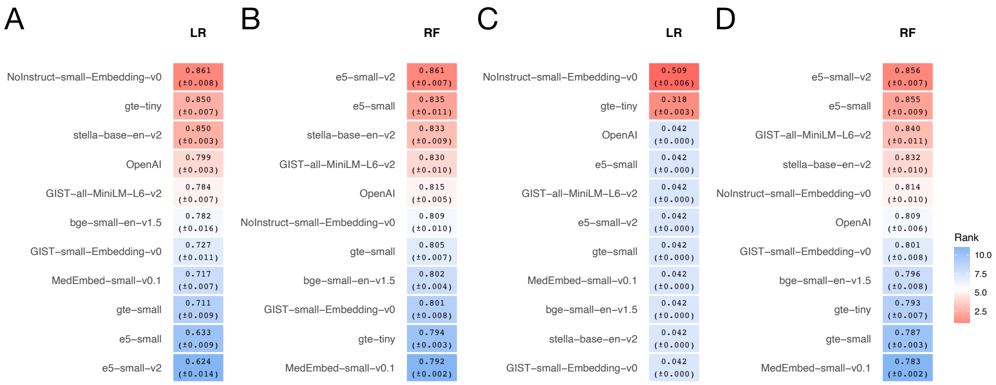

Figure S16: Performance of text embeddings on a large multi-class classification task. Panels A and B show results without hyperparameter tuning. Panels C and D show results with hyperparameter tuning.

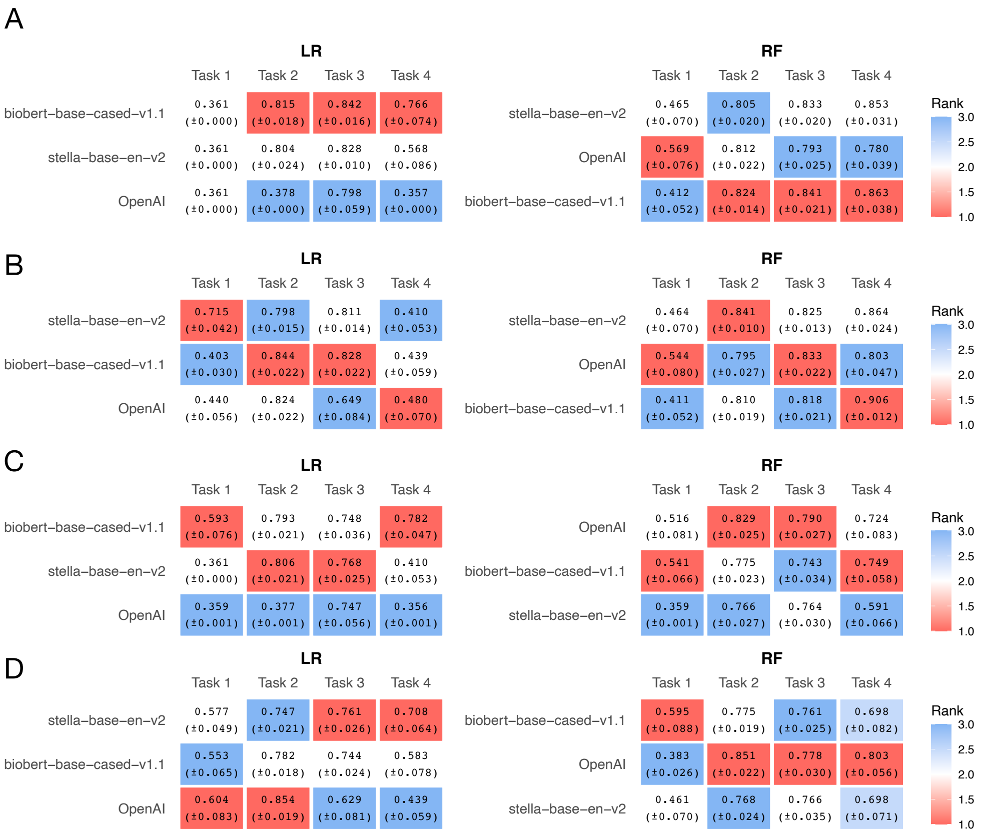

Figure S17: Effect of dimensionality truncation on model performance across four classification tasks. This figure compares the performance of three embedding models—OpenAI (1536 $\rightarrow$ 384), stella-base-en-v2 (768 $\rightarrow$ 384), and biobert-base-cased-v1.1 (768 $\rightarrow$ 384)—after truncating their embeddings to 384 dimensions. Panels A and B use text embeddings, while C and D use gene-symbol embeddings. Panels A and C show results without hyperparameter tuning, and panels B and D include hyperparameter tuning.

8 Recall

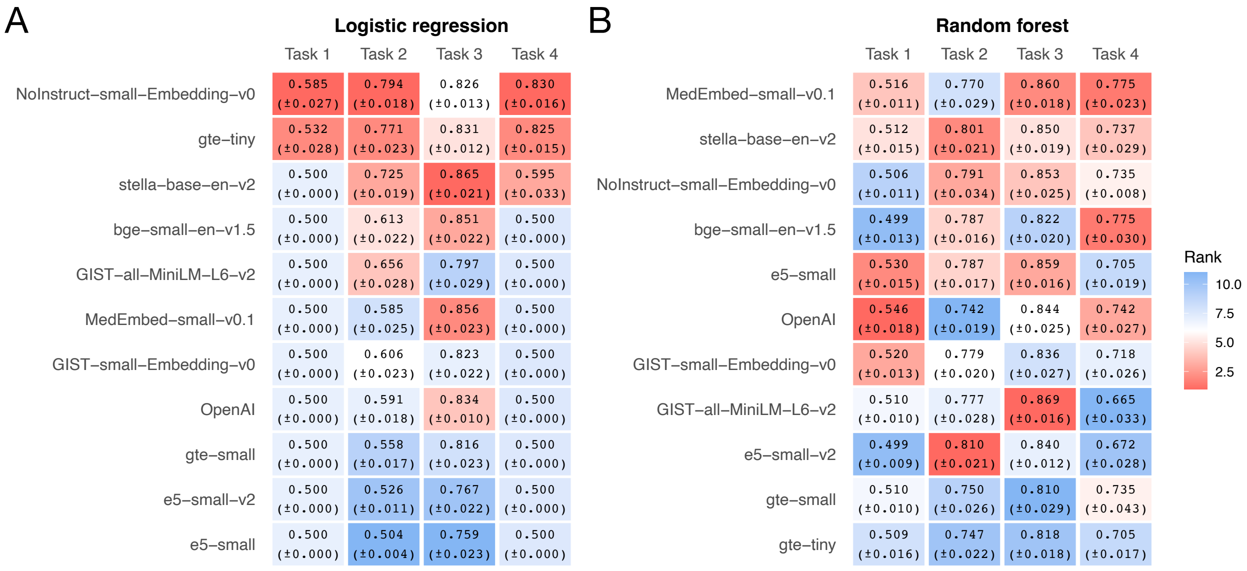

Figure S18: Classification performance across four tasks using gene embeddings derived from gene text descriptions without hyperparameter tuning.

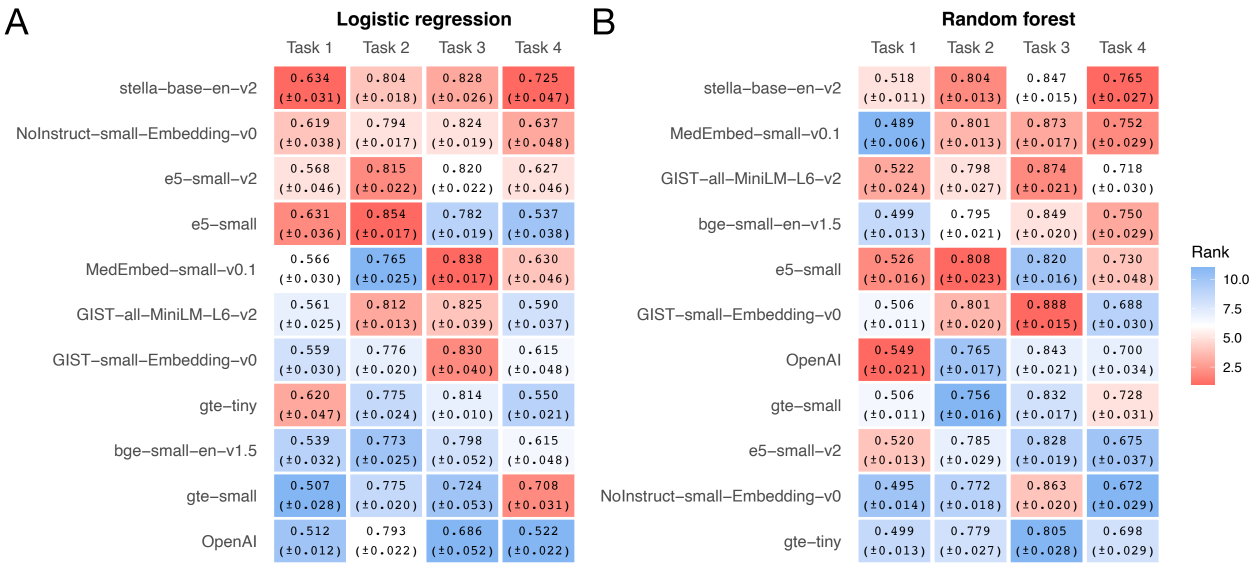

Figure S19: Classification performance across four tasks using gene embeddings derived from gene text descriptions with hyperparameter tuning.

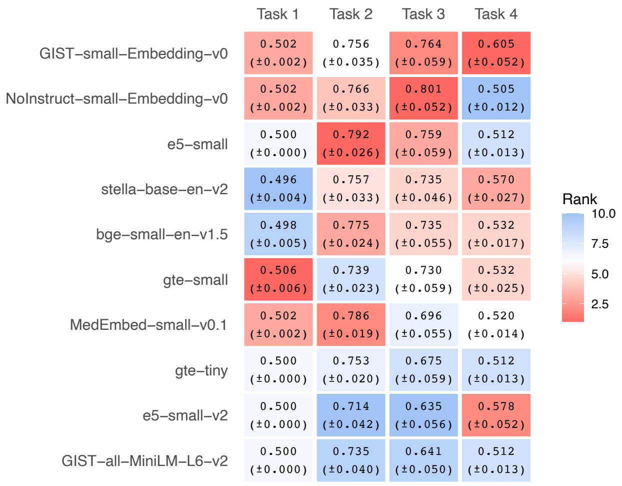

Figure S20: Fine-tuning performance across four classification tasks using gene text embeddings.

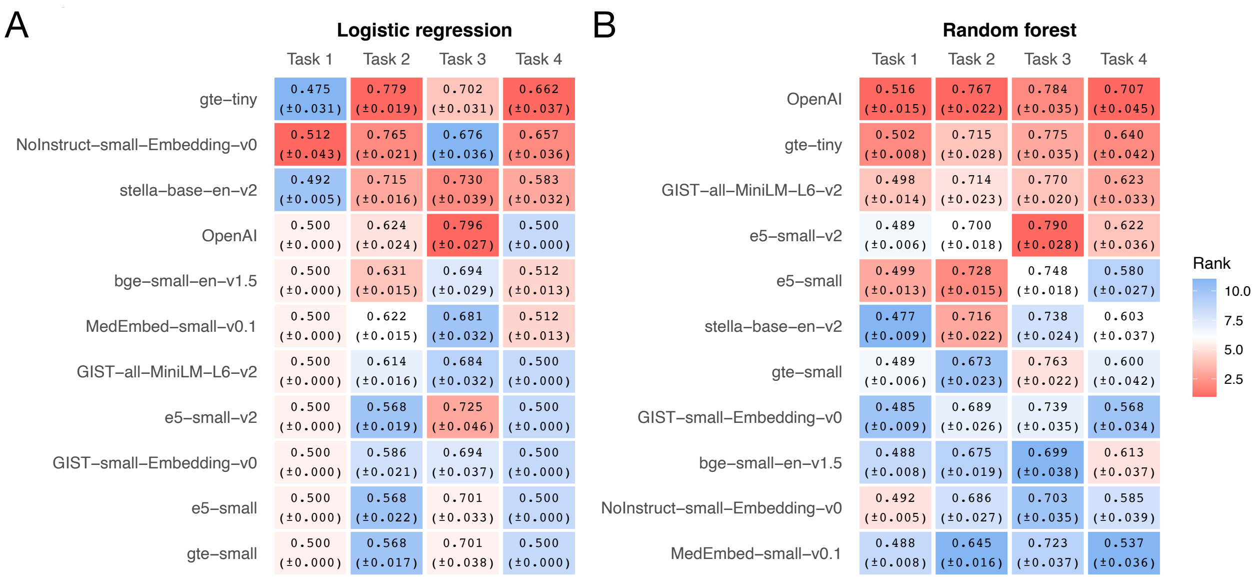

Figure S21: Classification performance across four tasks using gene embeddings derived from gene symbols alone without hyperparameter tuning.

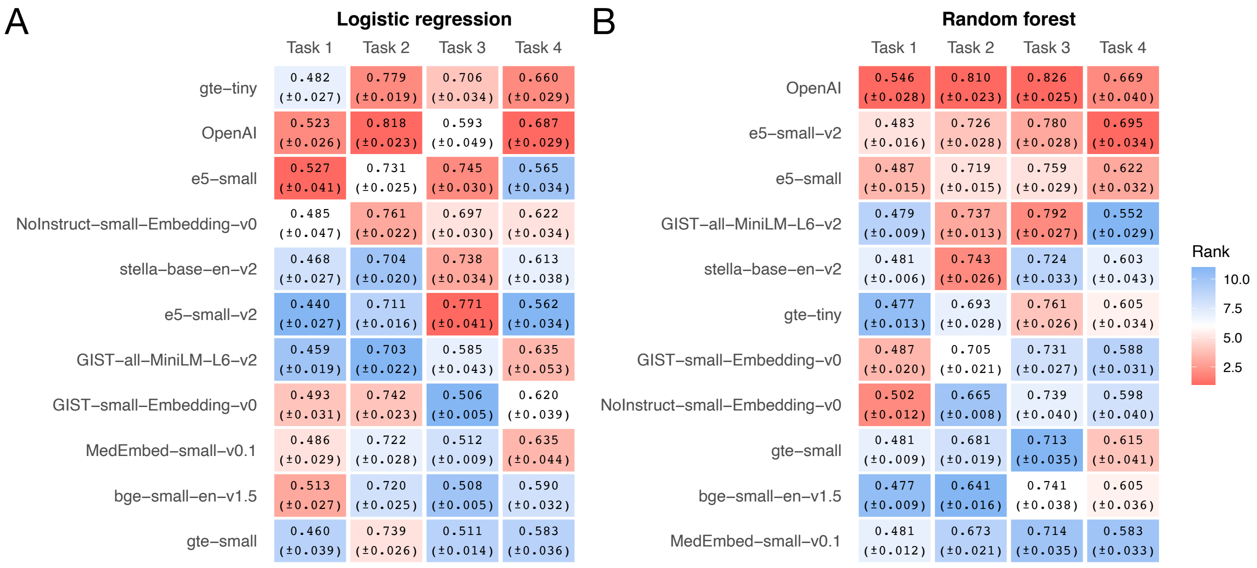

Figure S22: Classification performance across four tasks using sentence embeddings derived from gene symbols alone with hyperparameter tuning

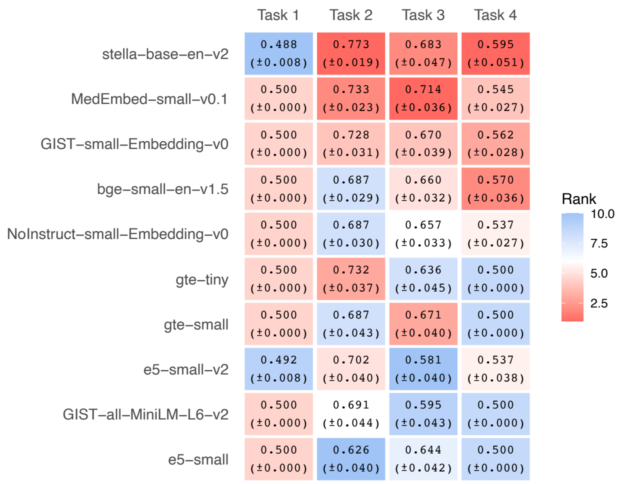

Figure S23: Fine-tuning performance across four classification tasks using gene symbol embeddings.

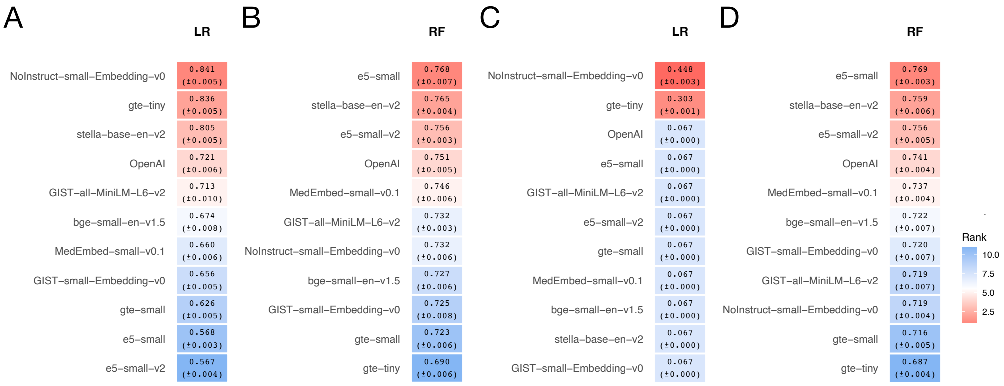

Figure S24: Performance of text embeddings on a large multi-class classification task. Panels A and B show results without hyperparameter tuning for logistic regression (A) and random forest (B), while panels C and D display results with hyperparameter tuning for logistic regression (C) and random forest (D).

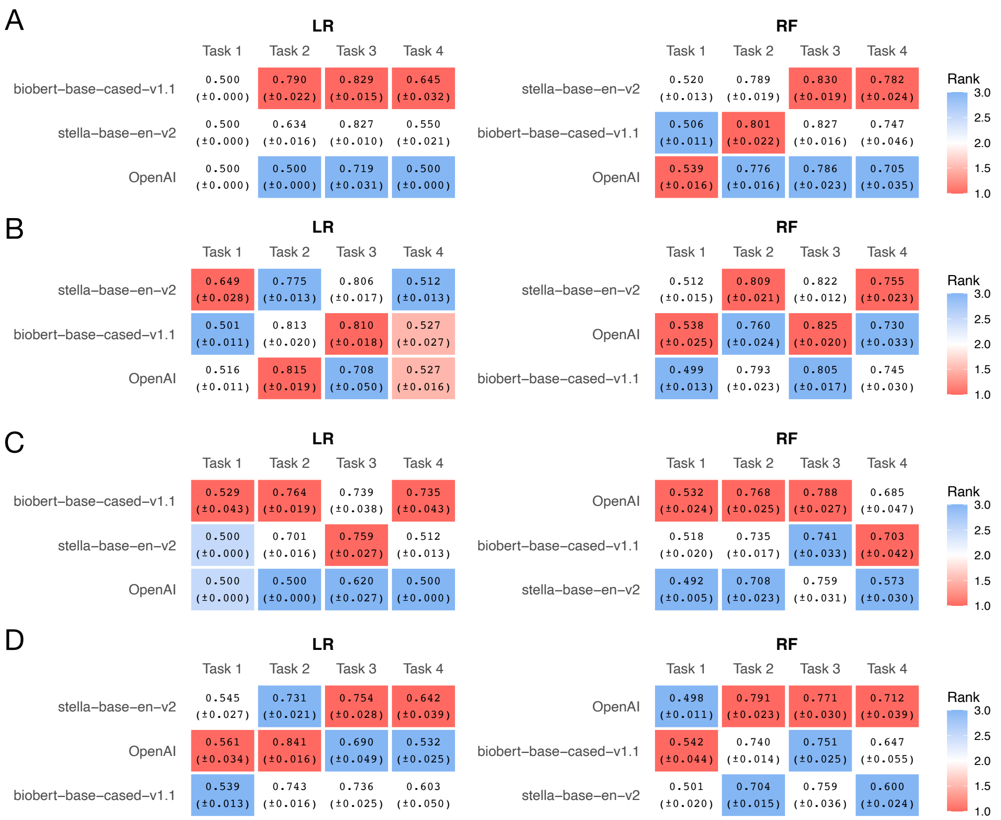

Figure S25: Effect of dimensionality truncation on model performance across four classification tasks. This figure compares the performance of three embedding models—OpenAI (1536 $\rightarrow$ 384), stella-base-en-v2 (768 $\rightarrow$ 384), and biobert-base-cased-v1.1 (768 $\rightarrow$ 384)—after truncating their embeddings to 384 dimensions. Panels A and B use text embeddings, while C and D use gene-symbol embeddings. Panels A and C show results without hyperparameter tuning, and panels B and D include hyperparameter tuning.

F1

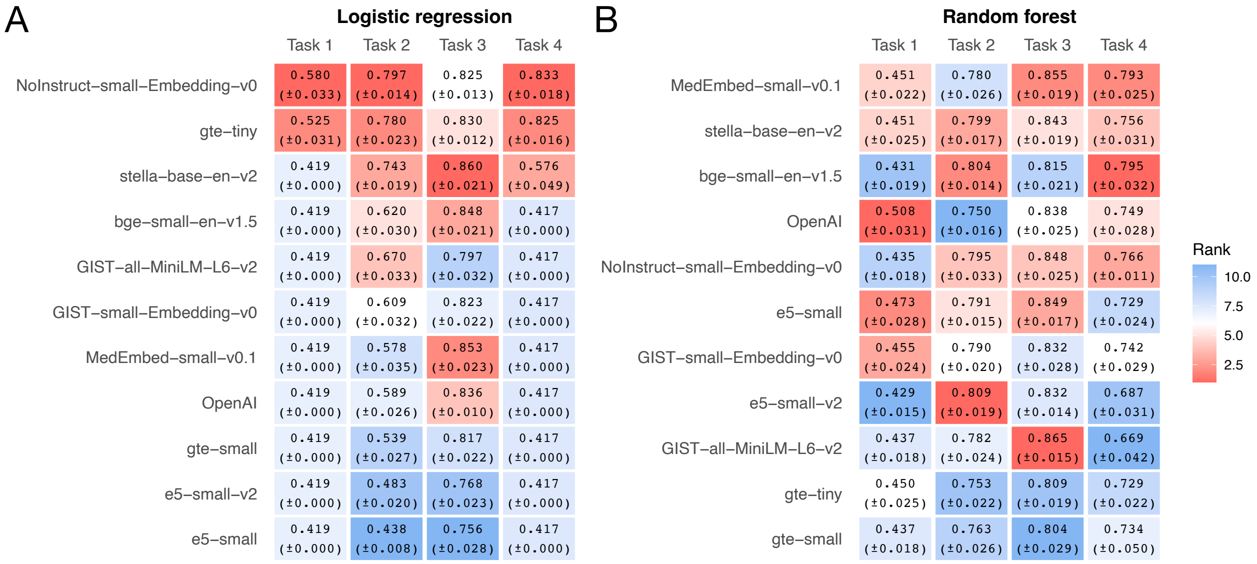

Figure S26: Classification performance across four tasks using gene embeddings derived from gene text descriptions without hyperparameter tuning.

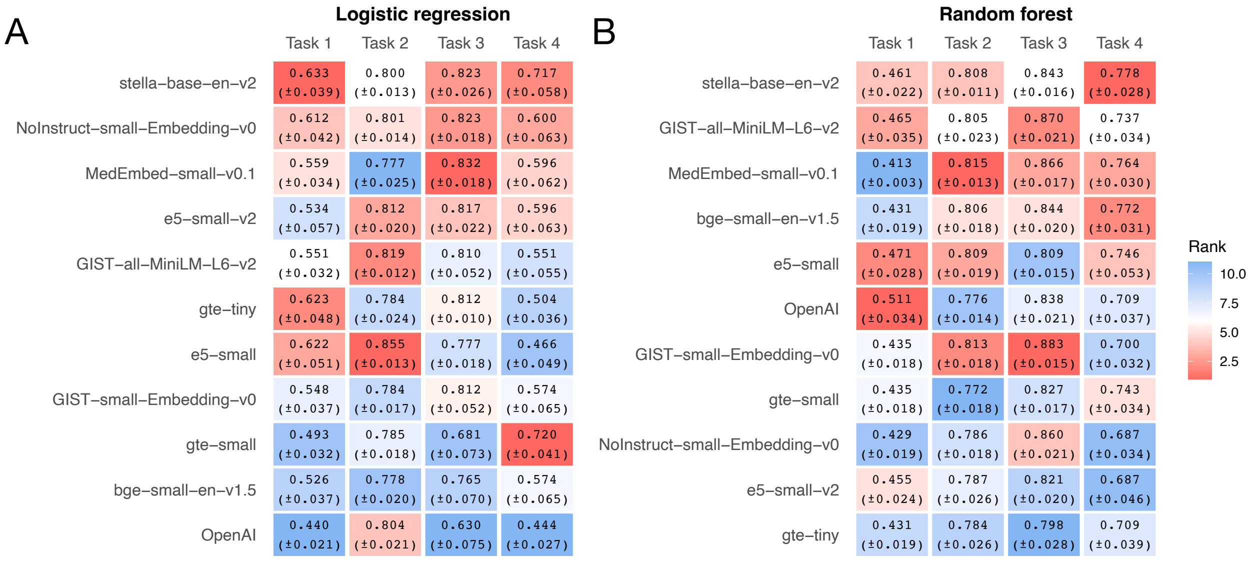

Figure S27: Classification performance across four tasks using gene embeddings derived from gene text descriptions with hyperparameter tuning.

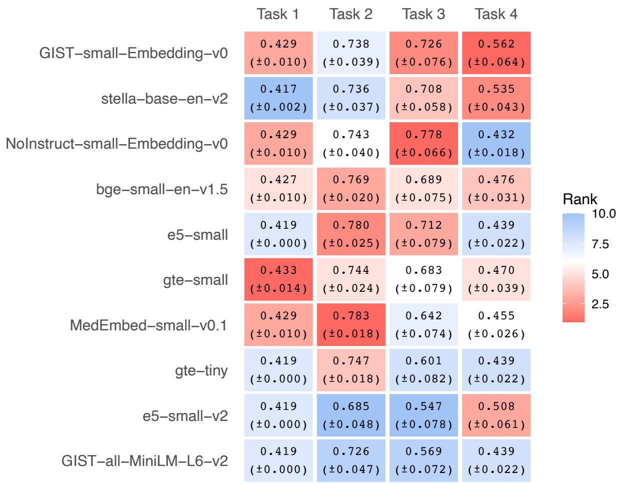

Figure S28: Fine-tuning performance across four classification tasks using gene text embeddings.

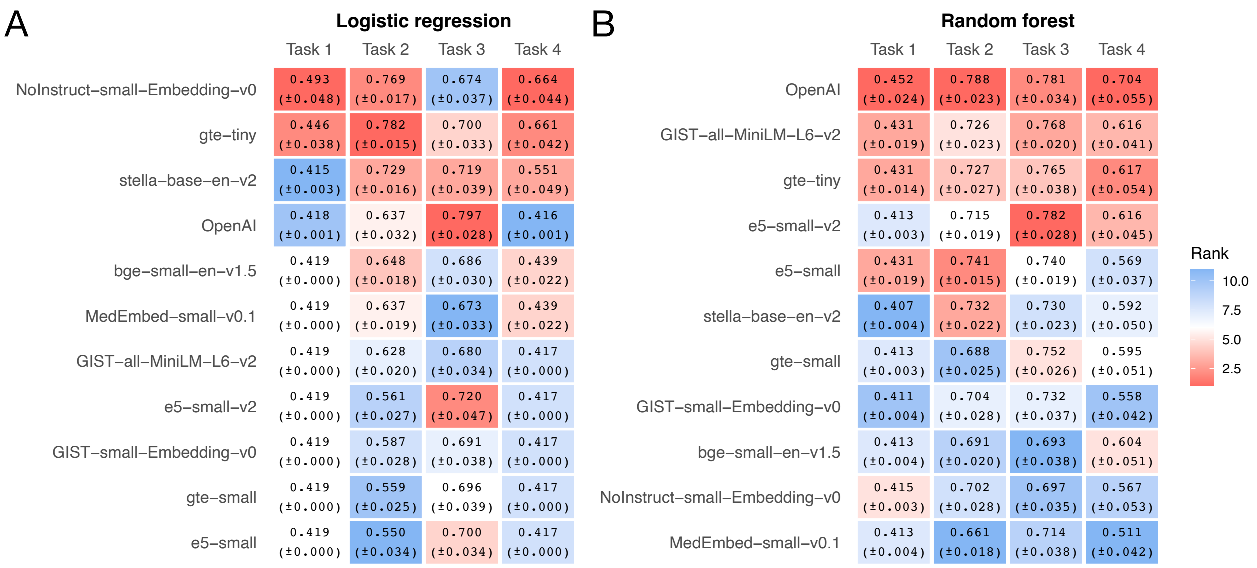

Figure S29: Classification performance across four tasks using sentence embeddings derived from gene symbols alone without hyperparameter tuning.

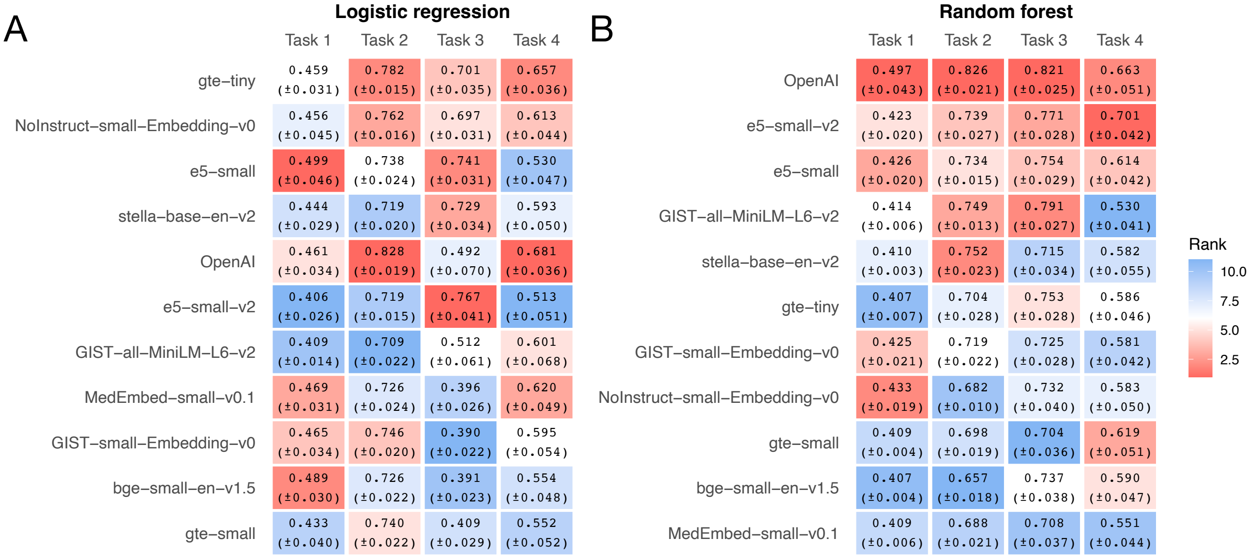

Figure S30: Classification performance across four tasks using sentence embeddings derived from gene symbols alone with hyperparameter tuning.

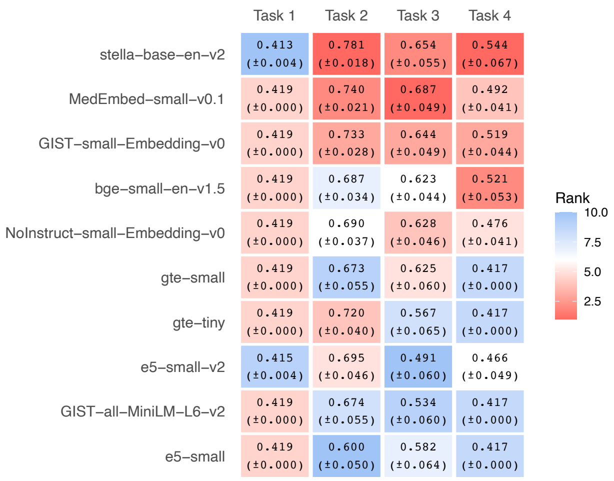

Figure S31: Fine-tuning performance across four classification tasks using gene symbols embeddings.

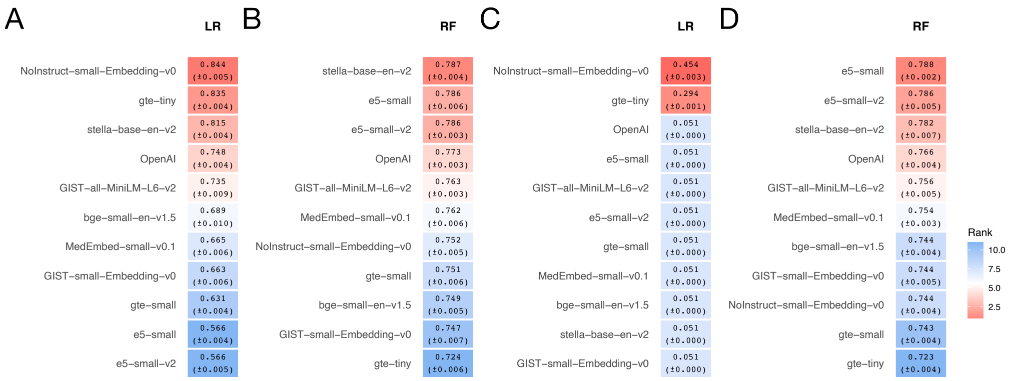

Figure S32: Performance of text embeddings on a large multi-class classification task. Panels A and B show results without hyperparameter tuning for logistic regression (A) and random forest (B), while panels C and D display results with hyperparameter tuning for logistic regression (C) and random forest (D).

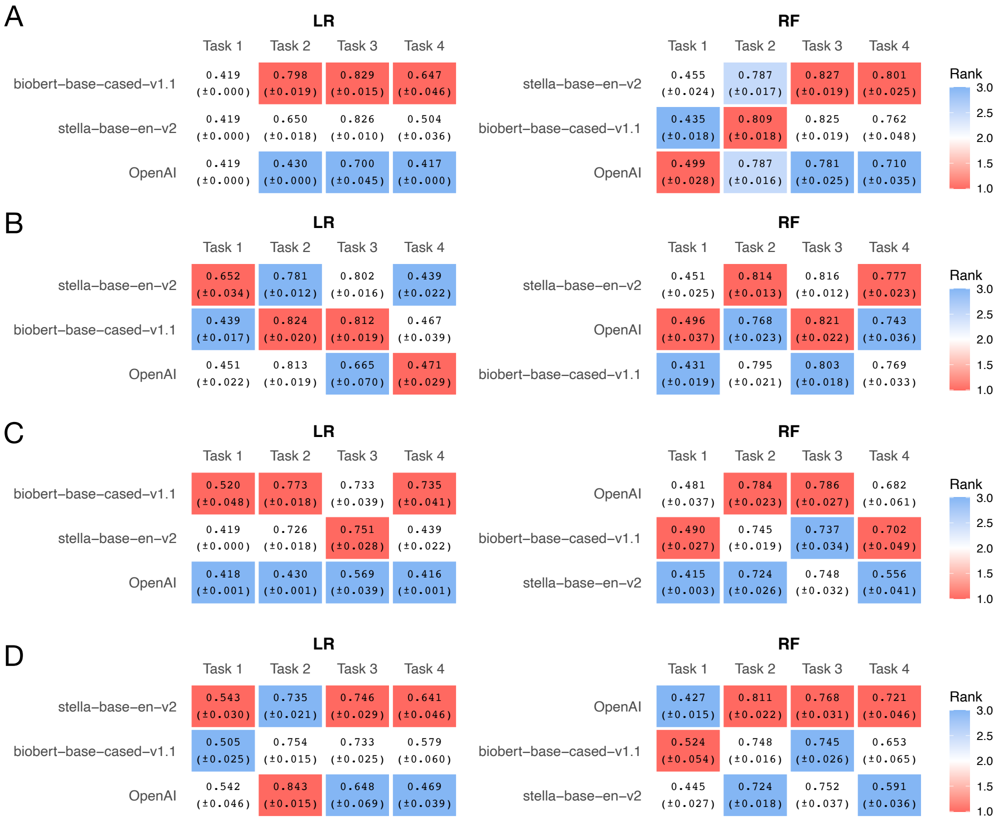

Figure S33: Effect of dimensionality truncation on model performance across four classification tasks. This figure compares the performance of three embedding models—OpenAI (1536 → 384), stella-base-en-v2 (768 → 384), and biobert-base-cased-v1.1 (768 → 384)—after truncating their embeddings to 384 dimensions. Panels A and B use text embeddings, while C and D use gene-symbol embeddings. Panels A and C show results without hyperparameter tuning, and panels B and D include hyperparameter tuning.
